# Social and Cognitive Interactions Through an Interactive School Service for RTT Patients at the COVID-19 Time

**DOI:** 10.3389/fpsyg.2021.676238

**Published:** 2021-06-24

**Authors:** Lucia Dovigo, Tindara Caprì, Giancarlo Iannizzotto, Andrea Nucita, Martina Semino, Samantha Giannatiempo, Lia Zocca, Rosa Angela Fabio

**Affiliations:** ^1^Airett Innovation and Research Center, Verona, Italy; ^2^Department of Clinical and Experimental Medicine, University of Messina, Messina, Italy; ^3^Department of Cognitive Sciences, Psychological, Educational and Cultural Studies, University of Messina, Messina, Italy; ^4^Tice Learning Center, Piacenza, Italy

**Keywords:** COVID-19, Rett Syndrome, telerehabilitation, attention, tele-health

## Abstract

**Background:** The closure of all educational institutions and most rehabilitation centres represents a precautionary measure to face the COVID-19 pandemic, but the isolation and social distancing may be particularly challenging for children with special needs and disabilities (SEND), such as Rett Syndrome (RTT). The main aim of this study was to promote cognitive and social interactions among children with RTT through an interactive school program.

**Methods:** The Interactive School palimpsest was composed of moments in which a teacher spoke directly to children with RTT and expected a response through eye gaze, and moments in which storeys-cartoon were presented while tracking the eye gaze of children. We investigated behavioural, social and cognitive parameters.

**Results:** Children participated in both social and cognitive tasks with the spontaneous reduction of stereotypies and with increase in attention. They recalled more significant indexes when music or a song was presented together with a cartoon or a cognitive task.

**Conclusions:** This study provides initial insights in promoting cognitive and social interactions and in the support needs of families with a child with RTT during the COVID-19 pandemic.

## Background

On 10th March 2020, schools, universities and all centres that hosted patients with special needs and disabilities (SEND) in Italy were closed to all pupils. This imposed closure of all educational institutions and most rehabilitation centres represents a precautionary measure to face the COVID-19 pandemic; similar measures have been taken in countries all around the world. Although this change was necessary, isolation and social distancing may be particularly challenging for SEND children and their families, given the reliance of many on carefully established routines and relationships, as well as professional and informal support (Toseeb et al., 2020; Zhang et al., 2020). For example, children with Rett Syndrome (RTT) constantly need to access equipment and professional support which is only available in rehabilitation centres. However, as RTT is a complex and rare disease, affecting 1:10,000 people, the management of this syndrome requires a high level of preparation and there are few specialised centres and specialists in Italy (Fabio et al., 2018a, 2020a). As a consequence, accessing adequate rehabilitation and special education treatments was particularly challenging for RTT patients, already before the COVID-19 pandemic, and the current imposed measures render conventional treatments even harder to access. In this context, life may become extremely difficult also for parents of SEND children, such as those affected by RTT, who meet their child's needs all day and every day, without the usual support of external professionals, teachers, schools and rehabilitation centres.

The use of technology to learn, live, and stay connected has become essential during a time of isolation and social distancing (Goldschmidt, 2020). Indeed, one of the most significant changes for millions of families and their child has been virtual schooling (Butcher, 2020). Public and private schools have created virtual platforms for virtual learning, but for SEND students, such as RTT, online learning has not been fully accessible (Kent, 2020). Hence, the closure of schools due to the COVID-19 emergency has caused SEND children and their families to experience serious educational problems (Narzisi, 2020; Yarimkaya and Esentürk, 2020). To our knowledge, no research exists addressing the question of how to promote social and cognitive interactions among SEND children during the COVID-19 pandemic; there are only general recommendations and suggestions (Gostin et al., 2020).

Supporting families with SEND children as affected by RTT is clearly necessary during the COVID-19 pandemic, and for this reason the Interactive School for RTT patients and their parents was created. The aim of the Interactive School was to bring the whole class or centre to RTT patients' homes through interactive teaching that motivated them to learn more, but, above all, to have fun being together with other children and teachers. The underlying rationale of this project is two-fold: technological and psychological. From the technological point of view, RTT patients suffer from severe difficulties in physical interaction, which in some cases leave them with only the ability to communicate and interact by means of their eye gaze (Fabio et al., 2018b). This requires technological support based on gaze tracking to allow for interaction with other people and with other technological platforms (Caprì et al., 2020a). From the psychological point of view, studies examining the effectiveness of multimedia technologies have demonstrated positive effects on the cognitive, communicative and motivational abilities of children with RTT (Fabio et al., 2018c, 2020b). In particular, it was found that multimedia presentations activate motivational factors which, in turn, increase the attention levels of patients with RTT. Attention is a multifaceted cognitive domain that can be divided into different functions such as “selective,” “shifting,” “sustained,” and “joint” attention. Selective attention refers to the ability to focus on the relevant or target stimuli; shifting refers to the capacity to disengage at one location and re-engage attention at a new location; sustained attention refers to the ability to maintain focus over an extended period; and joint attention refers to the ability to share a point of common reference with another person. With reference to the attention functions in RTT, Fabio et al. (2019a,b) for example, considered selective attention and evaluated the ability of patients with RTT to look at a target object in preference to other non-target objects or faces (of experimenter or caregiver). They also looked for evidence of joint attention behaviours, such as looking between target object and caregiver. Whilst identifying impairments in selective attention to target objects and a heightened interest in social stimuli, the authors found that selective attention to objects could be improved through containment of hand stereotypies and through training. Fabio and colleagues concluded that their results had important implications for communication and cognitive functioning, as increases in distractibility and in responses to irrelevant stimuli interfered with processing of relevant information. Rose et al. (2013) examined the selective attention of patients with RTT, as well as their ability to maintain attention, obtaining controversial results. In another study, Rose et al. (2016) found that children with RTT were capable of maintaining attention on a stimulus and can orient relatively quickly to the appearance of a target in the visual field. Instead, their recent study (Rose et al., 2019) showed that children with RTT demonstrated impairments in selective attention. Other authors found that children with RTT showed more difficulty with sustained attention when compared to a healthy control group (De Breet et al., 2019), and were slower to engage with moving objects, to be more distractible, and slower to re-engage. Due to this theoretical account of the attention functions in RTT, selective attention was the attention subcomponent involved in the tasks of the present study; fixation length was the measurement of the selective attention in this study.

Moreover, although RTT is a complex disorder characterised by loss of purposeful hand movements and speech, regression of acquired cognitive and motor skills, RTT patients do not have specific impairments in the theory of mind (ToM) ability and the teacher's face is an additional motivating factor in attracting their attention (Fabio et al., 2019a). From a clinical point of view, it was demonstrated that children with RTT show spontaneous non-verbal behaviours, such as gaze control and pointing, which are known to be precursors of ToM abilities and are not different from typically developing subjects (Antonietti et al., 2005). Research on ToM abilities in RTT (Antonietti et al., 2002, 2008) found that non-verbal training aimed at teaching basic and complex emotion recognition allowed two children with RTT to develop the ability to discriminate, recognise, and generalise the expression of emotions, which is considered to be crucially important for understanding the mental states of others. Moreover, it was demonstrated that children with RTT, if properly trained, were able to successfully perform a first and a second-order false-belief task, which is the most commonly employed and reliable task to evaluate the ToM ability (Castelli et al., 2013). In addition, children with RTT often remain visually attentive to objects and people, showing preferences for the human face and eyes, and they successfully use eye gaze to communicate (Vessoyan et al., 2018).

For all the aforementioned reasons, it was hypothesised that children with RTT can benefit from the possibility of social and cognitive school interactions through online systems, boosting their cognitive and social communication skills. The general objective of the present study was to evaluate the possibility of a virtual interaction between educators and patients with RTT, objectively measuring some outcomes in support of this possible interaction. Thus, the Interactive School for RTT was designed to catch RTT patients' attention and curiosity, and partly based on the communication platform previously developed for a tele-rehabilitation project (Caprì et al., 2020b). The specific objectives were to examine cognitive and behavioural interactions of patients with RTT during cognitive and social tasks and in the absence of a task. The Interactive School palimpsest was composed of moments in which the teacher spoke directly to the children with RTT and expected a response through eye gaze, and moments in which storeys-cartoon were presented while tracking the eye gaze of the children. We investigated:

- If the number of seconds of attention (fixation length) was higher on the cognitive or on the social task;
- If the time spent in stereotyping was higher in the absence of stimulation, in social and cognitive tasks;
- If the time of attention (fixation length) was higher on the teacher's face or other participants.
- If there was a correlation between fixation length and correct replies.

As RTT patients do not have deficits in ToM and the human face attracts their attention, we expected that the time of attention would be high both on cognitive and social tasks, that it would be also higher on the teacher's face than participants' face, and that the time spent in stereotyping would be lower when RTT patients were engaged in social or cognitive tasks than when in absence of stimulation.

## Methods

### Participants

Thirty-nine girls with RTT, ranging from ages 3–24 years (mean = 9.8 years) were recruited by the Italian Rett Syndrome Association (AIRETT). Demographic, developmental, clinical, behavioural, and genetic information was collected from all available sources such as parent/caregiver reports of past history, current behaviour and features, and latest clinical reports. Table 1 summarises the characteristics of all participants.

**Table 1:** Means (M) and (standard deviations) of characteristics of all participants.

**Characteristics**	**Values**
Number of participants	
Males/females	0/39
Age M (SD)	9.8 (4.7)
Stage III	20%
Stage IV	80%
RARS^a^ M (SD)	61.89 (2.40)
VABS^b^ M (SD)	89.91 (2.32)
Raven^c^ M (SD)	5.1 (3.45)

a *Rett Assessment Rating Scales (RARS)*.

b *Vineland Adaptive Behaviour Scales-Interview second edition (VABS)*.

c *Raven's Progressive Advance Matrices*.

### Technological Architecture

The Interactive School communication architecture leverages on the Cisco Webex conferencing system. The reason why we chose this system is that, at the time the Covid-19 emergency started, we were already using this platform for a telerehabilitation project (Caprì et al., 2020a). This timing coincidence helped us to immediately start with the Interactive School, since educators and therapists had already acquired skills to manage a videoconference with RTT children, though with a different purpose. In fact, during a telerehabilitation session the therapist supervises activities that are conducted locally by the patient with the help of a caregiver. On the contrary, during an Interactive School lesson, the educator administers several activities by means of multimedia material (such as presentations or videos) or asks for physical movements (e.g., play a tambourine). Then, RTT children have to respond to these stimuli, helped by the caregiver, and the interactions and levels of attention can be observed by means of an eye-tracker.

**Figure 1:**
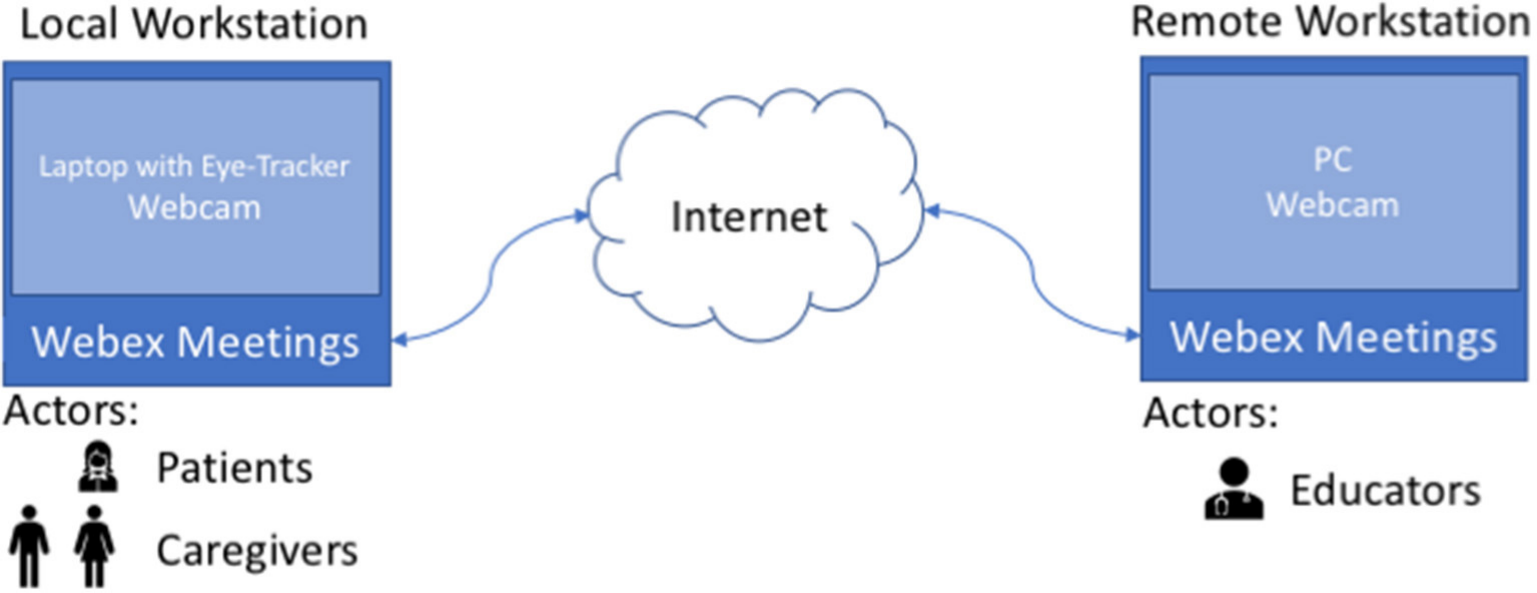
Interactive school architecture.

In Figure 1, the overall architecture is shown.

**Figure 2:**
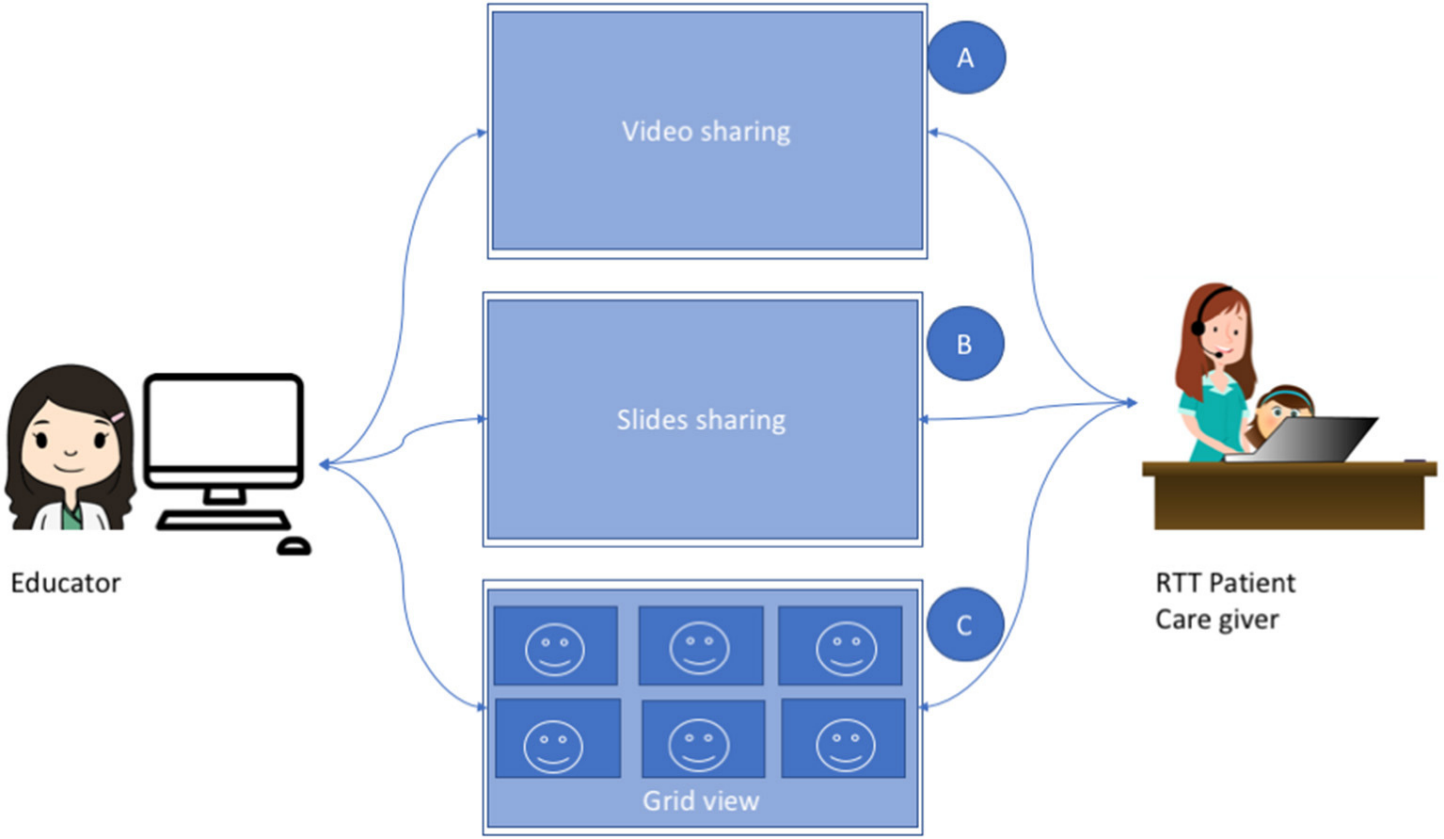
Lesson activities.

As already mentioned, during a lesson the educator administers multimedia material through the videoconferencing software. Multimedia material varies, each one requiring a different level of interaction by the RTT children. In Figure 2, we illustrate what is shown by the educator for each lesson activity. While the educator shares a video (e.g., a cartoon), each one of the connected RTT children sees the same content (Figure 2A). On the child's side, the interaction is acquired by the eye-tracker. Data on this interaction will be subsequently used for attention analysis. During the slide sharing, the educator shows some content and asks the children to answer a question, by choosing an image on the screen (Figure 2B). In this case, the caregiver in turn shares the screen with the educator, so that the educator can see the choice of the child by looking at the cursor on the screen, moved through the eye-tracker.

Finally, when interaction between the children is needed, the educator switches to grid view, in such a way that each participant can see the other participants. This occurs, for instance, when the educator asks the children to greet one each other (Figure 2C).

### Procedure

The Interactive School was composed of social and cognitive interaction intervals given some theoretical premises: multimedia presentations are motivational factors which, in turn, increase the attention levels of patients with RTT; these patients do not have specific ability deficits regarding the theory of mind (ToM); and the teacher's face is an additional motivating factor in attracting their attention (Castelli et al., 2013). With reference to the social interaction intervals, at the end of the opening multimedia presentation as outlined above, the teacher expanded the video of each participant in turn and invited them to introduce themselves according to their skills. With reference to the cognitive interactions, after the second social interaction, the video of a cartoon storey was presented. The cartoon changed in each section and it was extracted from famous animation movies, such as “Heide,” “Minnie,” “Mary Poppins” and calibrated according to the comprehensibility of the storey. Each cartoon lasted 2:30 min. At the end of the cartoon sequences, a recognition test was carried out for each participant. They were asked to perform, in turn, immediate recall of the cartoon with a recognition test composed of 10 questions regarding the storey. For each question, two pictures were presented on the screen, the correct answer and the distractor answer. The scoring standard used in the present study involved giving 1 point for choosing the correct answer, and 0 points for choosing the distractor. The total time of each session was about 20 min. The underlying rationale for the use of 2-choice design is based on the following factors. Firstly, subjects with RTT experience difficulties with communication, and only small proportions use words or gestures for communication. More commonly, these subjects use eye gaze to communicate. In addition, they have difficulties to processing more stimuli in the same times, because they have low reaction times. Secondly, the current literature does not provide a detailed description of choice making abilities of subjects with RTT. Nor does the literature adequately describe the relationships between choice making and factors known to influence other communication abilities such as MECP2 mutation type and the context of the communicative interaction. Thirdly, the application of binary format methodology is a practise that is supported by many researches and a number of empirical literature reviews on RTT.

Before starting the experiment, attention and stereotypies were measured in a without tasks or social engagement condition in which participants accessed the platform and waited for the start of experimental tasks for 2 min.

We carried out the experiment for 2 months. The Interactive School was structured as follows (see Supplementary Material):

- Who. A teacher, three or four patients with their caregivers connected online.
- When. The activity is carried out every day from Monday to Thursday, for about an hour, preferably at the same time (10.30 am).
- What. A 10-step lesson with multimedia and interaction activities.

### Data Analysis

*Eye-tracking*. Within each stimulus, a squared area of interest (AOI) around the target was defined. The size of the AOI covered a visual field of about 19 degrees.

For each AOI, relative to each stimulus, the fixation length (FL) was measured, which is the amount of time (seconds) spent by the girl when looking at the target. Fixations were extracted using a threshold of 100 ms.

### Measures

#### Behavioural and Cognitive Measures

With reference to the general behaviour of the patients with RTT the parameters were:

1. Number of seconds of attention (fixation length) to social and cognitive tasks for a maximum time of 10 min (600 s);
2. Time spent in stereotyping in the absence of stimulation, in social and cognitive tasks for a maximum time of 10 min (600 s).

#### Social Communication Task

**Figure 3:**
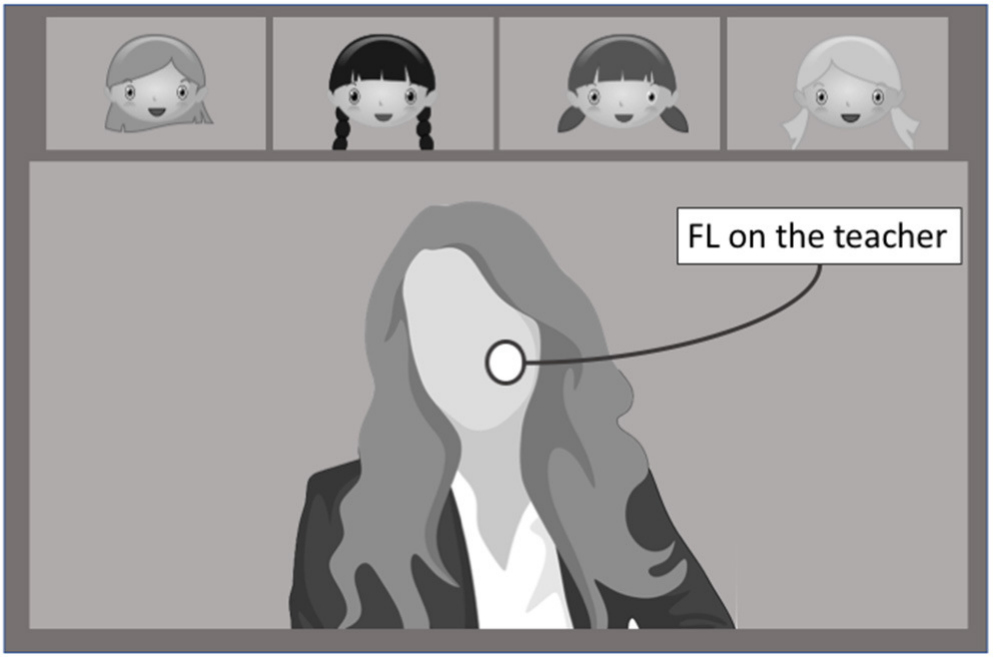
Computation of FL on the teacher.

With reference to social communication the parameters were:

1. FL of the participant on the teacher during the assignment of tasks or during reinforcements or singing a song (Figure 3);
2. FL of the same participant on the first girl that was invited to move or reply when she was called by the teacher;
3. FL of the same participant on the second girl that was invited to move or reply when she was called by the teacher;
4. FL of same participant on the third girl that was invited to move or reply when she was called by the teacher;
5. FL of same participant on the fourth girl that was invited to move or reply when she was called by the teacher.

#### Cognitive Tasks

With reference to cognitive tasks the parameters were:

1. FL of the participant on the main character of the cartoon;
2. FL of the participant on the PC screen but not on the main character;
3. FL of the participant outside of the PC screen;
4. Number of correct replies to the questions on the cartoon. The recognition test was based on 10 questions regarding the storey posed immediately after seeing the cartoon.

**Figure 4:**
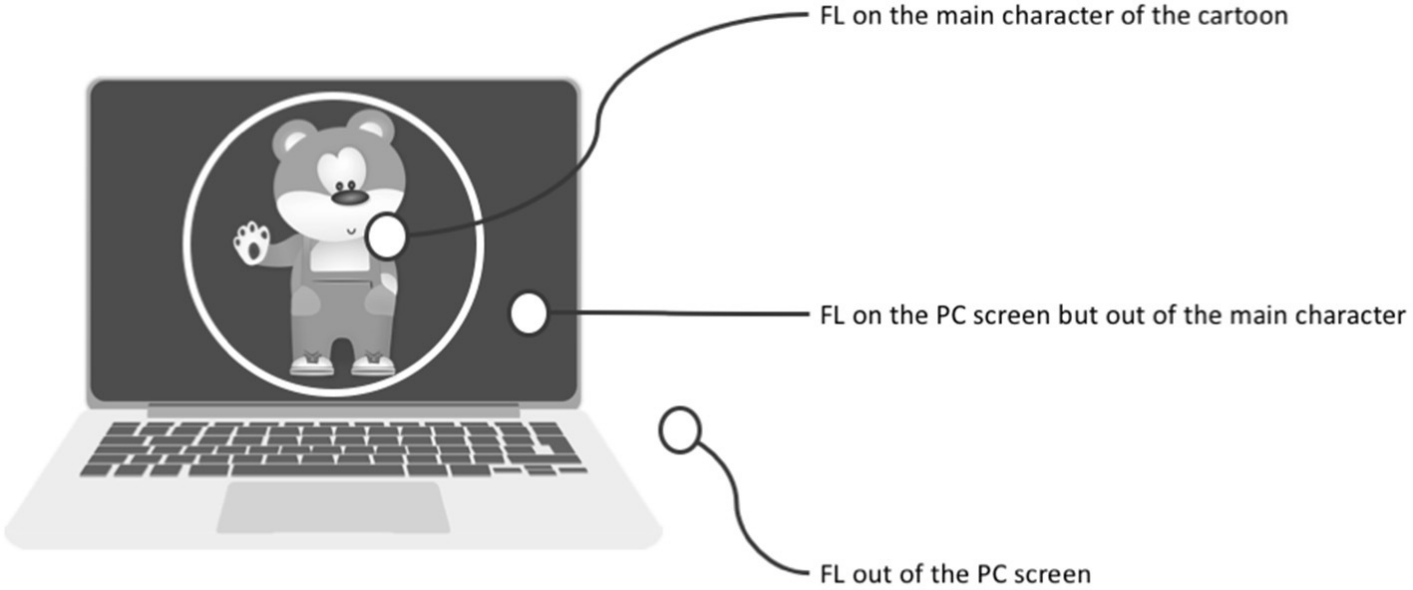
Computation of the three different FL.

More in detail, with reference to the cognitive task, FL was computed in the following way (Figure 4). In both tasks, FL refers to the amount of time (seconds) spent by the subject when looking at the correct stimulus. Fixations were extracted using a threshold of 100 ms.

### Statistical Analysis

Data were analysed using SPSS version 24.0 for Mac. The descriptive statistics of the dependent variables were tabulated and examined. Alpha level was set to 0.05 for all statistical tests. In the case of significant effects, the effect size of the test was reported. Data set, presented in the Supplementary Material, refers to the means of correct replies to the first movies presented each day.

To verify the effects of the considered variables in this study, the ANOVA repeated measurement design was carried out and Fisher's test was used. The relationship between variables was firstly evaluated by determining Pearson's r. Secondly, linear regression analysis was performed to evaluate the correlation between FL and correct replies (CR). The correlation coefficient β was used for linear regression analysis. The following guidelines proposed by Chan (2003) were used to assess the strength of the linear relationship: poor (β <0.3), fair (β 0.3–0.5), moderately strong (β 0.6–0.8), and very strong (β ≥ 0.8).

## Results

### Description of Participants

Thirty-nine girls with RTT, ranging from ages 3–24 years (mean = 9.8 years) were classified between clinical stage III and IV. Typically, the onset of symptoms begins with a severe regression around 18 months of age, and progresses through the three remaining stages of decline that include symptoms like the loss of purposeful hand skills and of spoken language (Stage II−1–4 years of age), seizures and apraxia (Stage III−4–10 years of age), and scoliosis and reduced mobility (Stage IV above 10 yrs. of age) (Amir and Zoghbi, 2000).

Due to the broad age range of participants (3–24 years) and two different clinical stages (III and IV stage), we subdivided the sample by age for median value (11.50) and by clinical stage (III and IV stage), so we examined the confounding effect of age and clinical stage using a preliminary ANOVA. This preliminary statistical analyses showed no significant effect for the above-mentioned parameters. This indicates that both age and clinical stages had no role in affecting the outcomes.

The results are first discussed with reference to behavioural and cognitive measures, secondly with reference to the social communication task and finally with reference to the cognitive task.

### Behavioural and Cognitive Measures

Regarding the number of seconds of attention (FL) to social and cognitive tasks, Table 2 shows the means and standard deviations of the parameters attention and time spent in stereotyping.

**Table 2:** Means and (Standard deviations) of the seconds of behavioural indexes.

	**FL in ss (attention)**	**Time spent in stereotyping**
No task		539.94 (56.12)
Social task	228.32 (210.21)	385.41 (76.20)
Cognitive task	264.32 (190.21)	360.21 (45.21)

With reference to the seconds of selective attention, the variable task (social vs. cognitive) shows significant statistical effects [*F*_(2,38)_ = 11.97, *p* < 0.0001]. As shown in Table 1, FL is higher in the cognitive task than in the social task. With reference to the seconds of stereotyping, the variable task (no task vs. social task vs. cognitive task) shows again significant statistical effects [*F*_(2,38)_ = 13.97, *p* < 0.0001]. As shown in Table 2, the stereotypes tend to be higher when the patients are not engaged than when they are engaged in a social task or a cognitive task (paired-t test is respectively t(36) = 8.1, *p* < 0.001 and t(36) = 7.21, *p* < 0.001). These results suggest that when patients with RTT are engaged in social or cognitive tasks they focus on them and reduce stereotyping behaviour; this means that multimedia presentations are motivational factors for subjects with RTT.

### Social Communication Task

With reference to FL of the participants on the teacher target and on the other participant targets, data were collected during the assignment of tasks, during reinforcements and during social phases such as “say hello,” “say goodbye” or “introduce yourself.” Table 3 shows the means and standard deviations of the FL parameters. Since social patterns vary in relation to context (a patient may need more time to look when called in a question or the teacher may speak for a longer time); pattern of attention and time spent are referred to in percentages (i.e., if the total speaking time of the teacher was 38 s and participant 1 looked at her for 38 s, 100% time was assigned; if participant 2 was requested to make a noise with an instrument for 12 s and participant 1 looked at her for 10 s, then 83.33% time was assigned).

**Table 3:** Means and (Standard deviations) of percentage of fixation length for each member of the interactive school.

	**Teacher**	**Participant 1**	**Participant 2**	**Participant 3**
Percentage of FL	55.58 (41.17)	47.22 (32.86)	33.16 (23.12)	36.59 (22.67)

The variable participants (teacher, participant 1, participant 2, participant 3) shows significant statistical effects [*F*_(3, 90)_ = 4.97, *p* < 0.01]. More specifically, the participants tend to pay more attention to the teacher than to the other participants. This means that the teacher's face captures more the attention of patients with RTT than other participants face.

### Cognitive Task

With reference to cognitive tasks, the parameters were again FL on the main character of the cartoon (on the main character, on the PC screen but outside of the main character and outside of the PC screen; see Figure 1) and the number of CR to the 10 questions regarding the storey of the cartoon.

The variable Attention (to the main character, away from the main character but on the screen, outside of the screen) shows significant statistical effects [*F*_(2,76)_ = 37.97, *p* < 0.001]. More specifically, the participants tend to pay more attention to the main character of the cartoon than away from it or outside of the screen. However, if only cartoons containing songs are considered, the time spent in looking at the main character was significantly higher (almost 100% of FL). This indicates that auditory stimuli, such as songs, are significant motivational factors for patients with RTT.

Moreover, the percentage of CR to the 10 questions on the storey of the cartoon was very high: 68% (with means and standard deviation 6.8 and 2.39). This performance showed high correlation with FL (Table 4). These results indicate that both multimedia presentation and human faces significantly capture the attention of patients with RTT.

**Table 4:** Means and (Standard deviations) of percentage of fixation length for the cartoon.

	**Main character of the cartoon**	**Out of the main character on the screen**	**Out of the screen**
Percentage of FL	76.38 (27.01)	6.12 (7.86)	15.86 (14.12)

### Correlational Analysis

R Pearson correlation measurement was applied to verify the correlation between FL and CR (Table 5).

**Table 5:** Pearson correlations.

	**CR**	**Main character of the cartoon**	**Out of the main character on the screen**	**Out of the screen**
CR	1	0.37^*^	−0.128	−0.55^**^
Main character of the cartoon		1	−0.35^*^	−0.88^**^
Out of the main character on the screen			1	−0.046
Out of the screen				1

** *p < .001*,

* *p < .05*

The correlation between FL of the target stimuli and CR of the stimuli is very high, *r*(40) = 0.37, *p* < 0.011, and is also consistently inversely related to fixation outside of the screen, *r*(40) = −0.55, *p* < 0.001. Since the correlations were statistically significant confirming an association between FL and CR, regression coefficients were applied to determine the role of FL as a predictor of CR value. Linear regression analysis showed that the correlation between FL and CR was strong (β = 0.78, *p* < 0.01). Also, the correlation between FL of fixation outside of screen and CR was strong, but inversely related (β = −0.73, *p* < 0.01, respectively). These results indicates that FL predictes the accuracy of response. More precesily, looking inside of the screen was associated with a better accuracy of response, whereas, looking outside of screen was associated with a worse performance. This means that direction of eye-gaze represents a potential indicators of expected performance in patients with RTT, confirming that RTT patients' pattern of preferential looking can be used as an indication of interest of patients with RTT (Djukic et al., 2012; Schwartzman et al., 2015; Ahonniska-Assa et al., 2018).

## Discussion

The objective of the present study was to evaluate the possibility of a virtual interaction between educators and children with RTT, objectively measuring some outcomes in support of this possible interaction. As described in this paper, the Interactive School was composed of social moments in which the teacher spoke directly to the children with RTT and expected a response, and moments in which different cartoon storeys were presented while tracking the eye gaze of the children, followed by a recognition test. We examined behavioural, cognitive and social skills of the participants in terms of number of seconds of attention to social or cognitive tasks, duration of attention to the teacher or the other participants.

As expected, participants attended both social and cognitive tasks with spontaneous reduction of stereotypes and with increased attention. They recalled more significant indexes when music or songs were presented within the cartoon or the cognitive task. Also, we found that when children with RTT were engaged in social or cognitive tasks, they attended to them and reduced their stereotyping behaviour. These findings confirm results presented in literature, demonstrating that children with RTT show a high level of attention and reduce their stereotypes if correctly stimulated (Fabio et al., 2009, 2011, 2018d). They also confirm the preference of children with RTT for human faces, as in this study, we found that the participants tended to attend more to the main character of the cartoon than away from it or outside of the screen and they focused more time on the teacher's face than on the other participants. As regards correlation analysis, Pearson's coefficient r was chosen as the measure of correlation strength. The relationship between FL and CR was very high. This result suggests that when the attention of children with RTT is correctly focused and captured by tasks, the number of CRs increases.

This study supports the idea that the children with RTT can benefit from the use of technology-aided programs, such as teleconference platform plus cognitive and social tasks, to promote cognitive and social skills. However, the present study has some limitations that are related to the sample size, the use of technologies, the specific historical period due to the Covid-19 pandemic. The sample size is small and there may be constraints to the generalizability of the results. However, the effect size is adequate; consequently, the results can be considered reliable and should be validated by a larger sample size. Moreover, it is important to consider that these technologies are subject to some limitations, because even though they have universal design features, patients with RTT present heterogeneous and complex impairments. As a consequence, technology must be matched to the RTT profile to meet the individual's needs (Davies et al., 2018). As regards the historical period, in these days, we are facing the worst and most challenging scenario we have ever dealt with, the Covid-19 pandemic. The rapid spread of the disease has led Italian institutions to resolutely invite the population to stay at home, especially people whose health conditions are frail. Children with RTT are thus confined to their homes, as every other person, but with a much heavier burden because of their condition and the lack of specific school programs. As a consequence, parents become the only ones who can stimulate the attention of RTT children and care for them, thus they carry a huge caregiving burden and their psychological stress and/or anxiety increase. It is probable that RTT children can experience the anxiety or stress related to parental burden and this can have a significant impact on both the lives of parents and child with RTT.

In accordance with previous studies (Aruanno et al., 2018; Damianidou et al., 2018; Iannizzotto et al., 2020) we suggest that more accessible online education tools should be designed to consider a wider range of concerns, such as feature of the syndrome, age and online environment. Moreover, the lack of specific interaction technologies, such as eye tracking may sometimes hinder the effectiveness and the availability of the proposed approach, as such technologies may be unavailable in specific cases (entry-level or mobile computing platforms). For such cases, lightweight, widely compatible eye-tracking software modules should be made available, which can be easily installed and seamlessly integrated with teleconferencing software to produce a reasonably reliable replacement for the hardware tracking device (Pramuka and Roosmalen, 2015; Tozzi et al., 2015; Getz et al., 2016; Jafni et al., 2017; Peretti et al., 2017).

This study has implications for both public health, parents and professionals. This research may be useful in terms of alleviating the caregiver's burden and, during the Covid-19 pandemic, through the Interactive School program involving both RTT patients and their caregivers, providing easy access to treatment services. Furthermore, the Interactive School program can be used by those who do not necessarily have the specialised knowledge of steering and prompting children with RTT. This means to significantly contribute to the long-term sustainability and profitability of healthcare systems, in terms of cost savings, improved patient care, and reduced workload to clinicians and caregivers. As in the current global situation due to the pandemic of Covid-19, this study demonstrates that this program can effectively deliver interventions to RTT patients without the risk of transferring the virus to human healthcare personnel. Thus, policy makers of public assistance healthcare could consider the use of teleconference platforms as a valid solution in providing treatment services during future lockdown or precautionary measures to face the Covid-19 pandemic.

However, given the complexity of RTT, it is necessary to develop specific validation approaches through well-designed evaluation processes, to confirm the benefits and potential effects of technology-mediated, and remotely administered, social and cognitive tasks for patients with RTT. In our future studies, we better describe the eye-tracker and stereotypes analysis, we will used interviews and questionnaires for parents and will examine their responses.

## Conclusion

This study aims at paving the way towards a more integrated and systematic application of telerehabilitation for RTT, introducing a novel online approach to rehabilitation that professionals can exploit to enhance and simplify their treatment programs. The proposed approach directly addresses one of the major issues in RTT rehabilitation, i.e., continuity. It has been experimented during the Covid-19 pandemic, when RTT children could not meet the caring professionals in person and allowed them to pursue their rehabilitation objectives timely and smoothly. Thanks to the wide availability of the needed hardware and software infrastructure (albeit there was a lack of specific interaction technology), the flexibility of the technological platform and the effectiveness of the rehabilitation methods, the proposed approach could reach widely distributed children in a capillary way, including those who had the most severe difficulties in reaching the professional caregivers in person. Finally, even for those patients who could receive their treatments in person, telerehabilitation services dramatically reduced the need for periodic in-person meetings, thus allowing the professionals to take care of more patients and reducing the cost for the families.

The present findings also shed new light on how parents of children with RTT would like to be supported during the Covid-19 pandemic. In particular, this study suggests that teleconference platforms can be used both in daily living and typical clinical settings and that they have a positive impact on many levels of functioning, from cognitive to social. We argue that the availability of the presented services will enable an innovative approach to rehabilitation for RTT, reducing social costs while widening the access to professional caring services for those families with logistic or economic difficulties.

To summarise, this study presented an innovative approach to telerehabilitation based on widely available teleconferencing technologies, that proved to be effective and efficient, while significantly reducing the social costs and improving the continuity of treatment for RTT patients.

## Data Availability Statement

The datasets presented in this study can be found in online repositories. The names of the repository/repositories and accession number(s) can be found at: tcapri@unime.it.

## Ethics Statement

The studies involving human participants were reviewed and approved by The Ethics Committee of the University of Messina. Written informed consent to participate in this study was provided by the participants' legal guardian/next of kin.

## Author Contributions

LD and RF: conceptualization and supervision. RF: methodology. AN and GI: software. LD: resources. TC, MS, and SG: data curation. RF, TC, AN, and GI: writing—original draft preparation. TC and RF: writing—review and editing. MS, TC, LZ, and SG: project administration. All authors have read and agreed to the published version of the manuscript.

## Conflict of Interest

The authors declare that the research was conducted in the absence of any commercial or financial relationships that could be construed as a potential conflict of interest.
