# Supplementary material for: Social and Cognitive Interactions Through an Interactive School Service for RTT Patients at the COVID-19 Time
Source: Front Psychol. 2021 Jun 24;12:676238. doi: 10.3389/fpsyg.2021.676238 (PMC8265204; doi:10.3389/fpsyg.2021.676238)
Supplement: Supplementary file 1 [file Data_Sheet_1.docx]

Supplementary materials

Questions of recognition test

| 1.Who was the main character of the story? |
| --- |
| 2. Who met princess at the beginning of the story? |
| 3. What colour was princess’s dress? |
| 4. What was princess doing? |
| 5. Was princess sad or happy? |
| 6. Were there clouds or stars? |
| 7. Who the princess played with?  8. How the princess played?  9. What colour were princess’s hair? |
| 10. What did princess do at the end of the story? |

Example of the structure of the Interactive School for RTT subjects.

|  | Lesson step | Activity | Description |
| --- | --- | --- | --- |
| 1 | Opening theme | Show multimedia  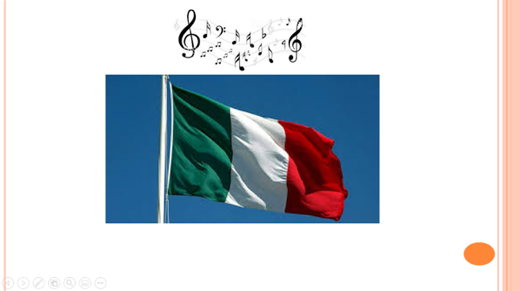 | An opening multimedia is shown, in our example a video with the Italian National Anthem |
| 2 | Greetings from the teacher | Webcam view with teacher in full screen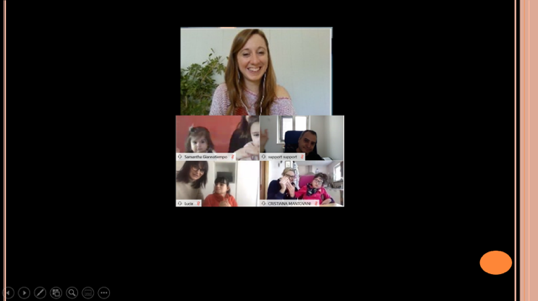 | At the end of the opening multimedia, the teacher introduces herself |
| 3 | Greetings among the girls | Webcam view with a split view among participants  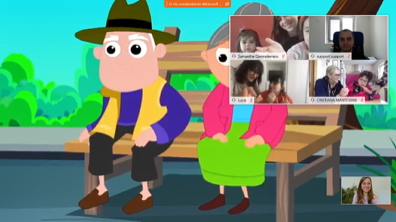 | The teacher expands the video of the girl and invites participants to introduce themselves according to their skills. The caregiver can help her daughter during this social interaction, for example, she/he can raise the girl’s hand. |
| 4 |  | Video presentation  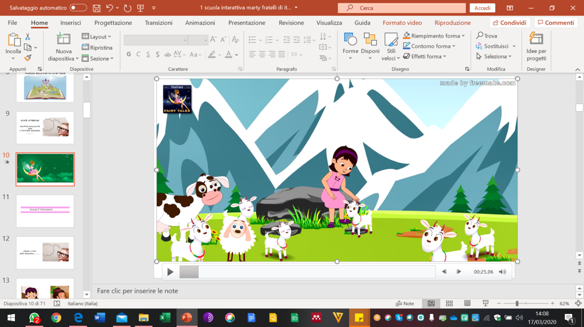 | After initial greetings, the video of a fairy tale is presented. The tale changes in each section. |
|  |  |  |  |
|  |  | Questions  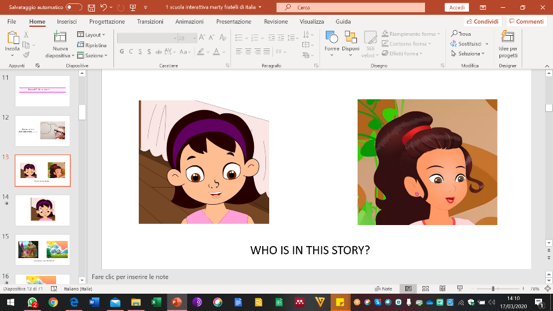 | Multimedia with a fairy tale and following the questions  Note:  *During this viewing it will be important for the teacher to have feedback of the girls' faces to draw attention if necessary or reinforce them. Binary multiple-choice answers with recognition ability is the answer of students with Rett Syndrome* |
| 5 | Game of perceptual synchronization (sounds that are repeated, visual imitation games) with video connection. | Webcam view with a split view among participants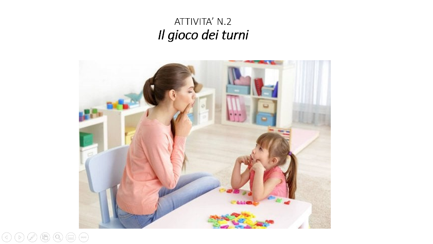 | The teacher proposes an imitation game to each girl, for example: the teacher can play once time the drum, so she invites a girl to repeat the same action with her tool or object. This game is repeated for each girl. |
| 6 | Closing greetings from the teacher | Webcam view with teacher in full screen  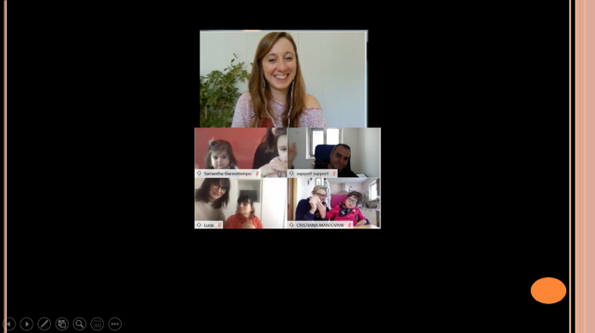 | The teacher thanks participants. |
| 7 | Closing greetings of the girls to each other | Webcam view with a split view among participants  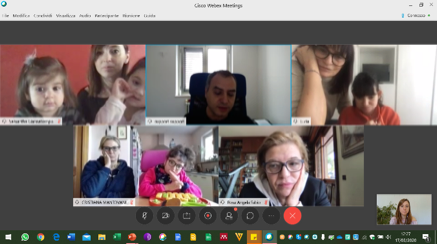 | Each girl greets others. |
