# Supplementary material for: Social and Cognitive Interactions Through an Interactive School Service for RTT Patients at the COVID-19 Time
Source: Front Psychol. 2021 Jun 24;12:676238. doi: 10.3389/fpsyg.2021.676238 (PMC8265204; doi:10.3389/fpsyg.2021.676238)
Supplement: Supplementary file 3 [file Presentation_1.pptx]

## Slide 1
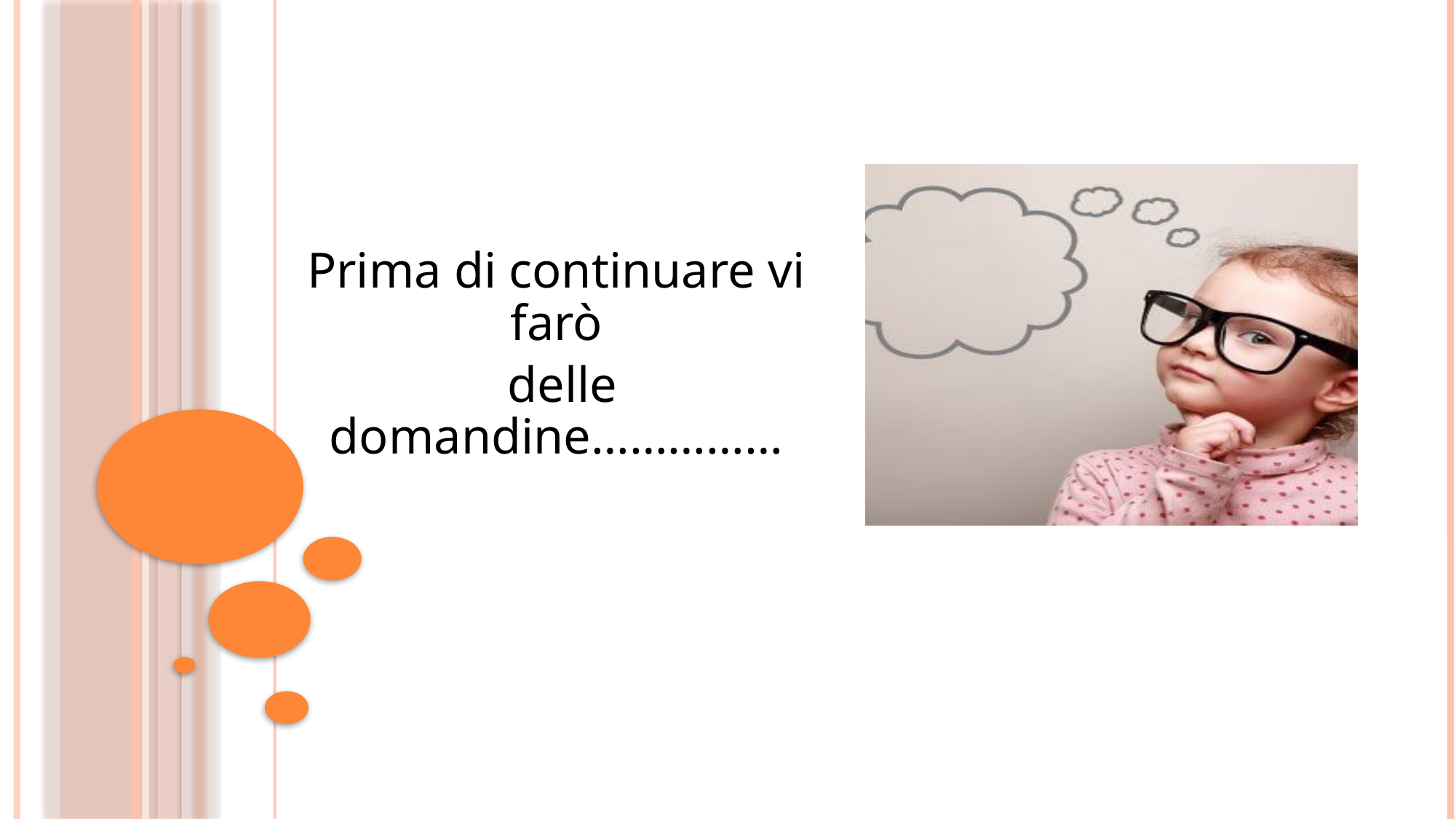

Prima di continuare vi farò
 delle domandine……………

## Slide 2
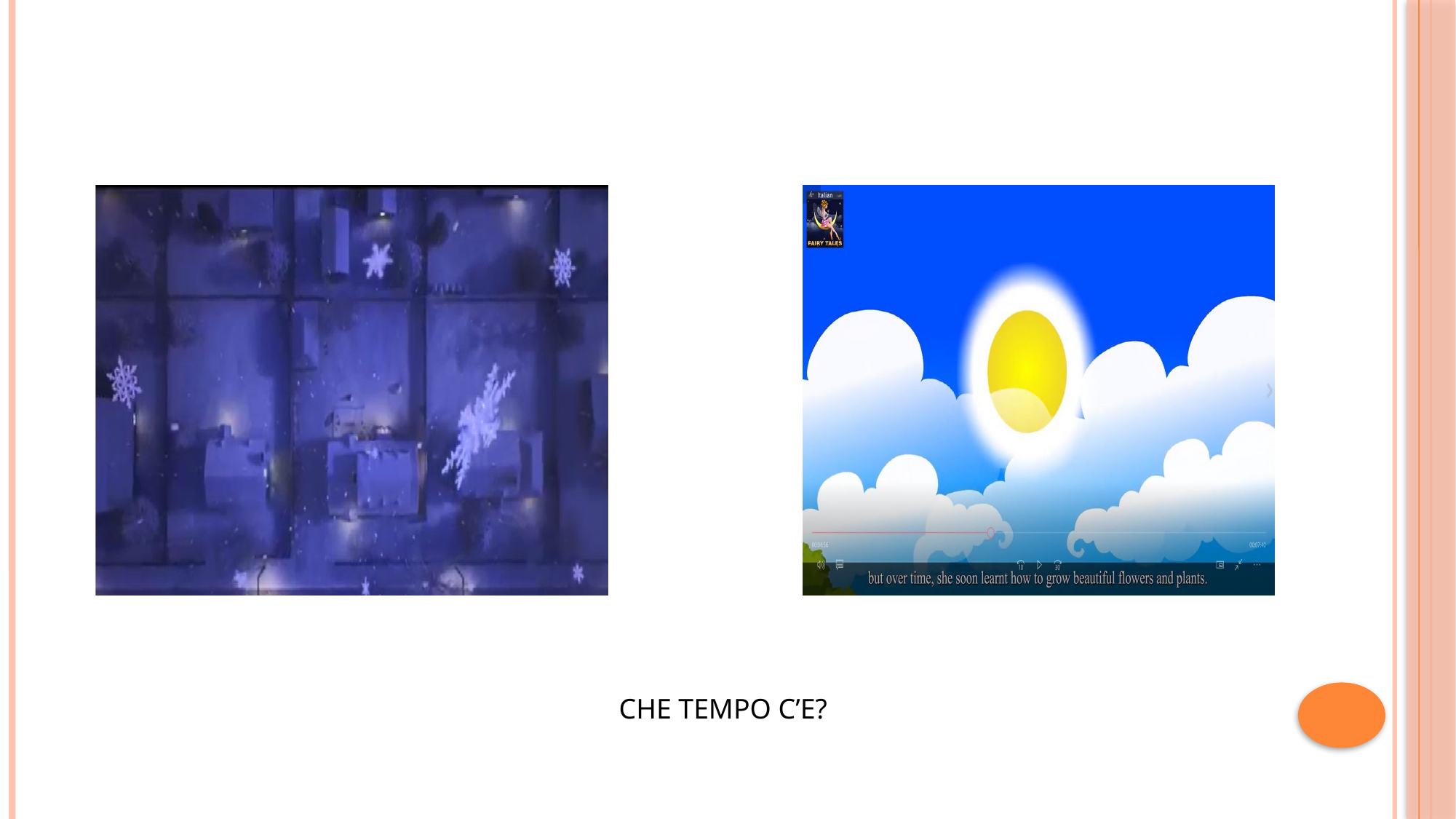

CHE TEMPO C’E?

## Slide 3
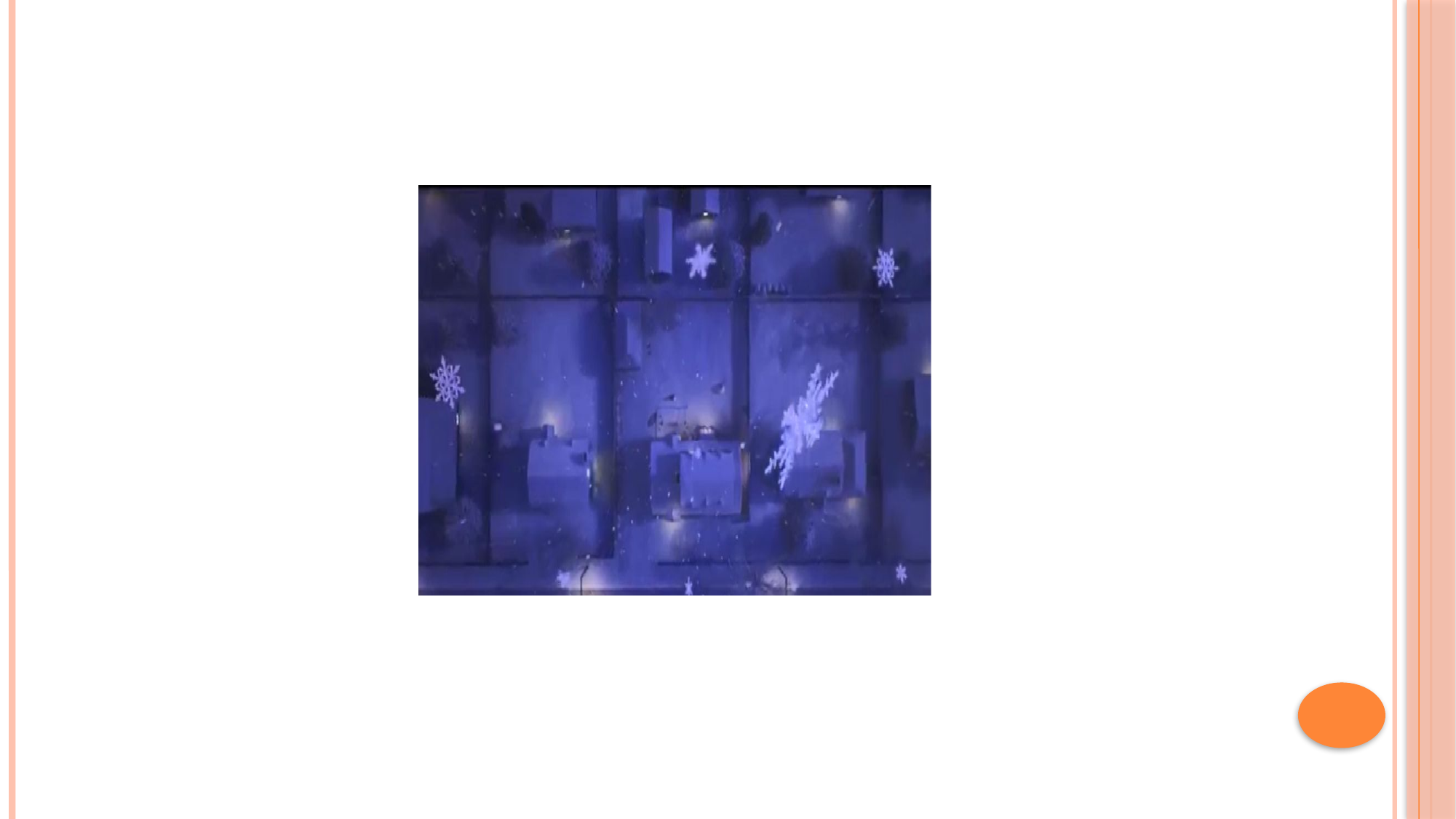

## Slide 4
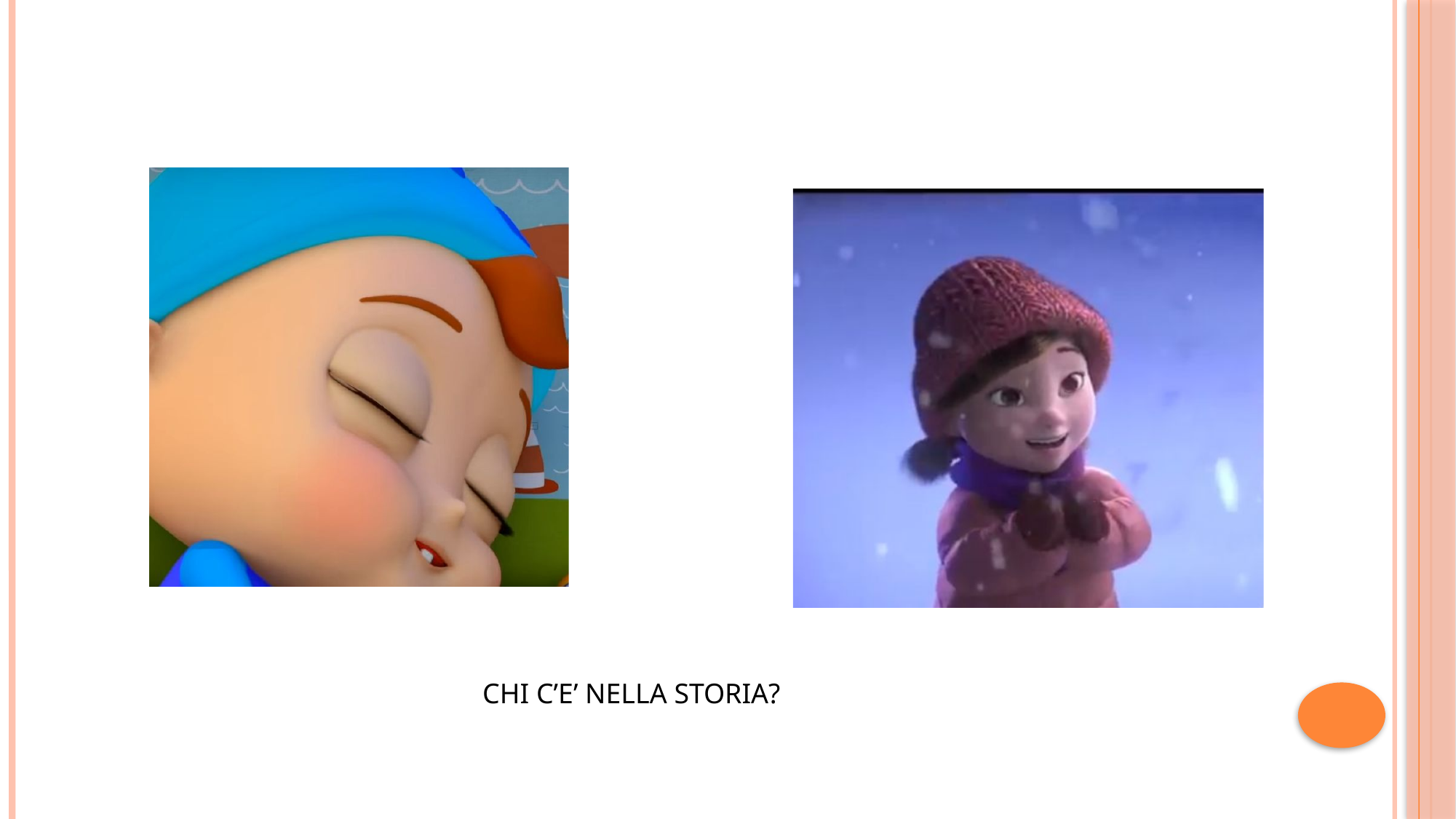

CHI C’E’ NELLA STORIA?

## Slide 5
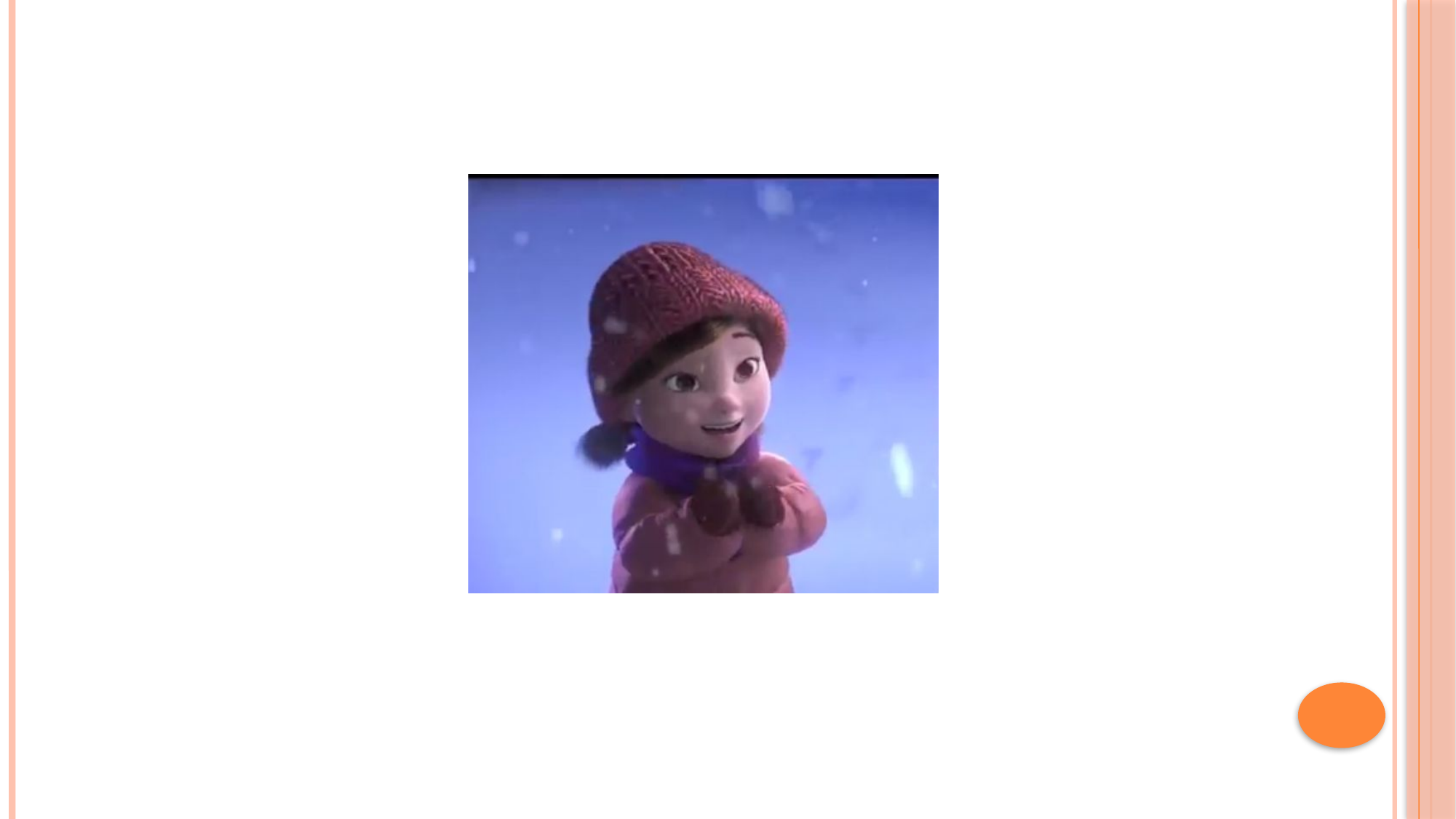

## Slide 6
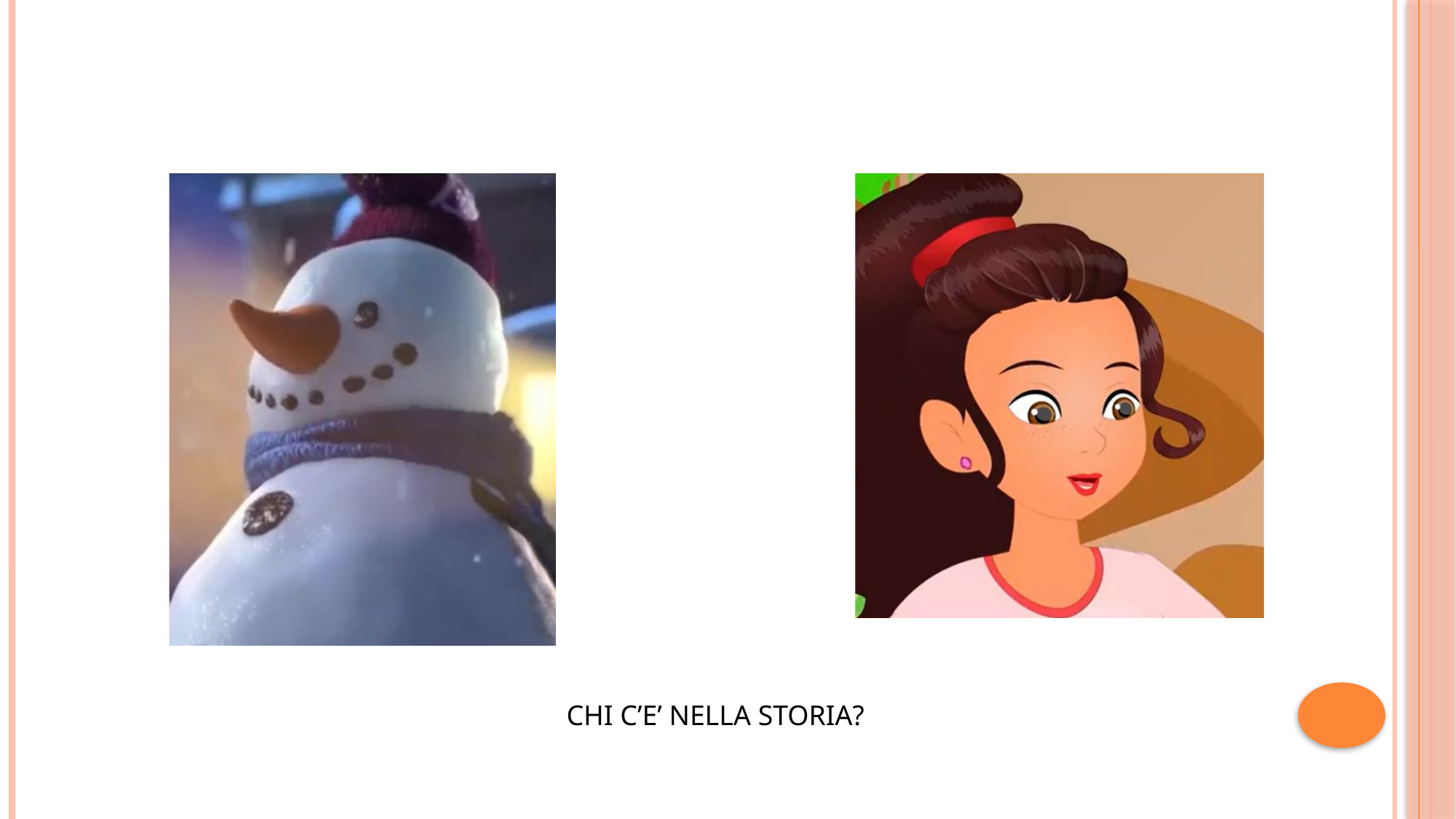

CHI C’E’ NELLA STORIA?

## Slide 7
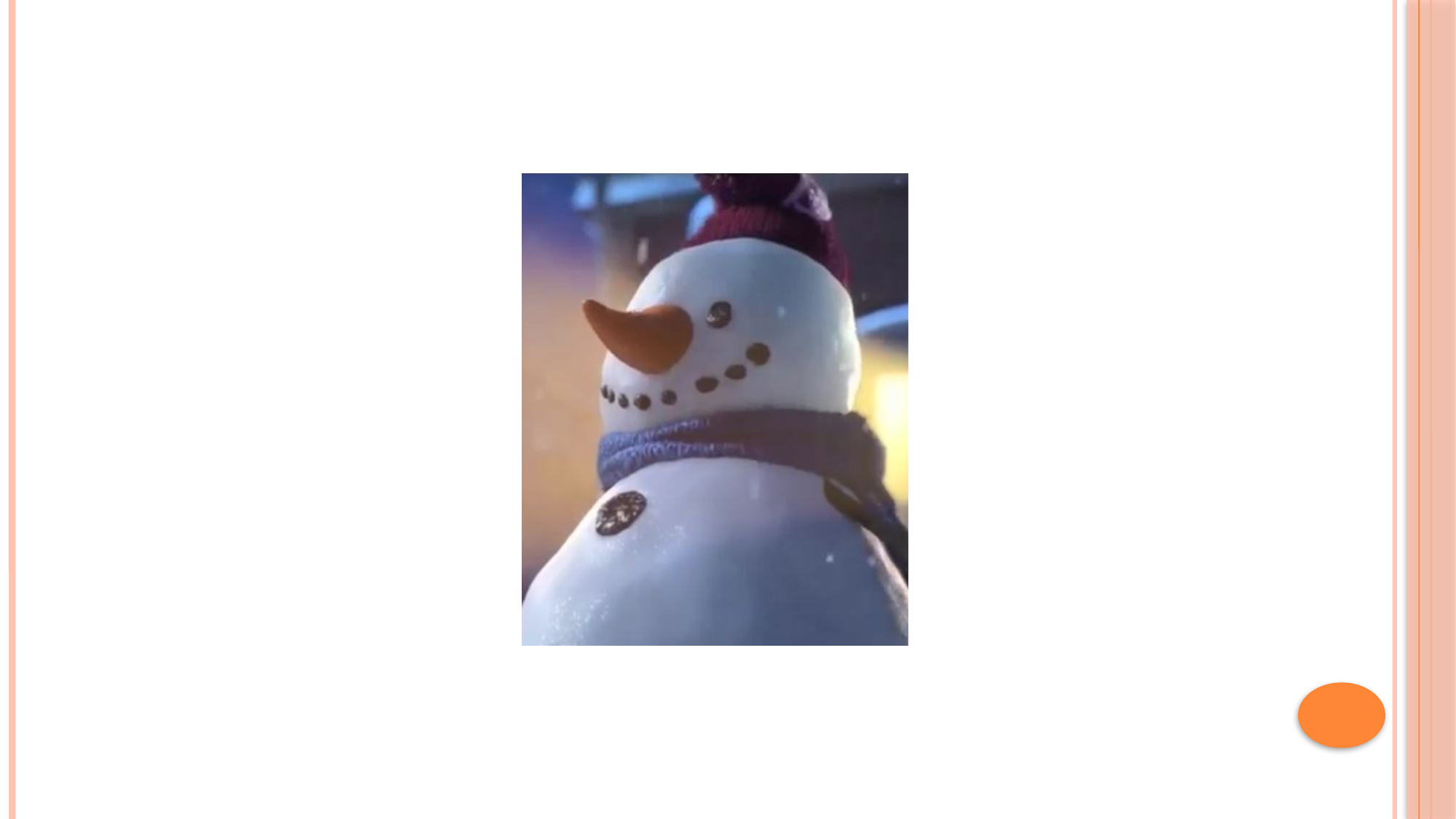

## Slide 8
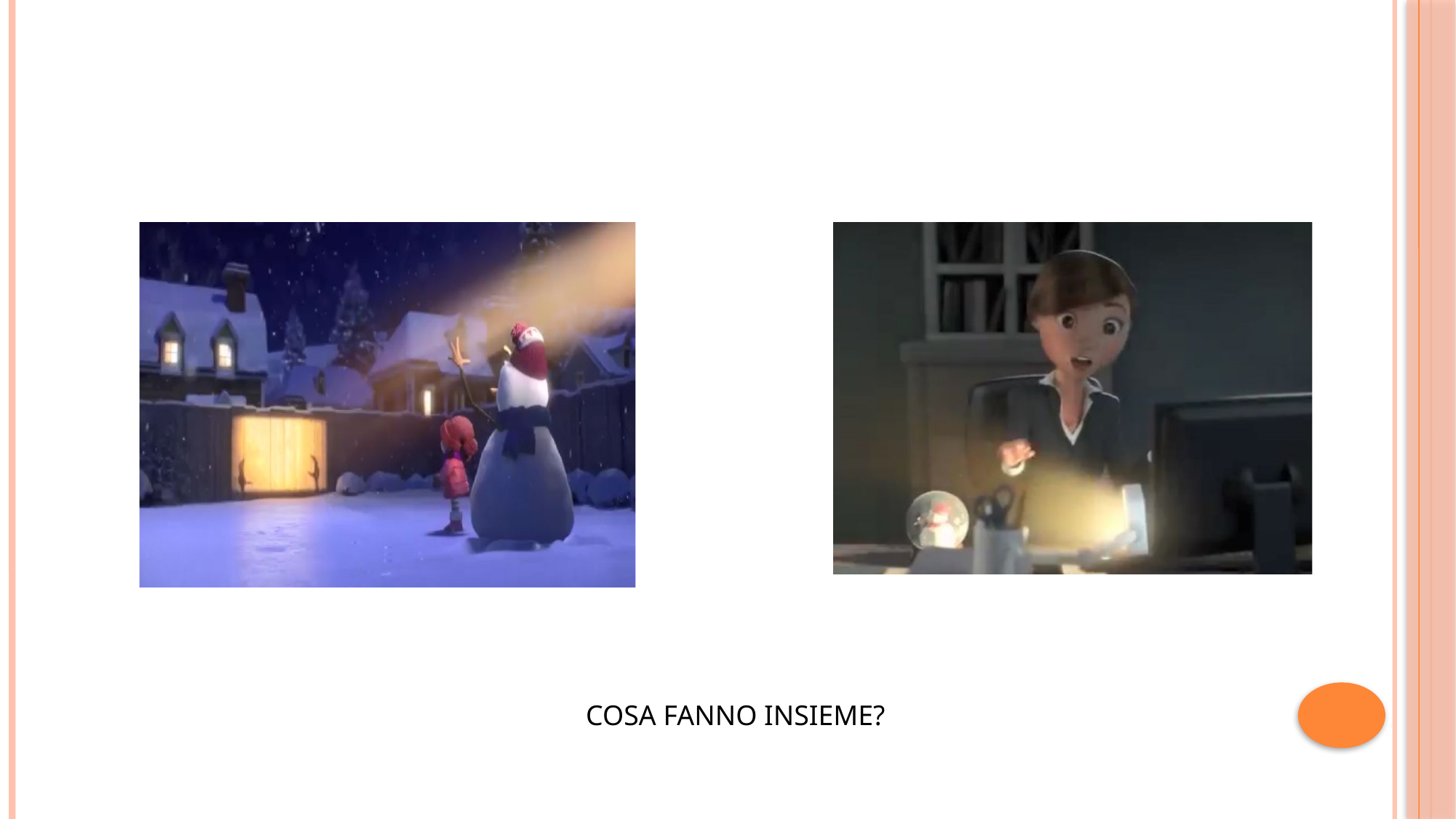

COSA FANNO INSIEME?

## Slide 9
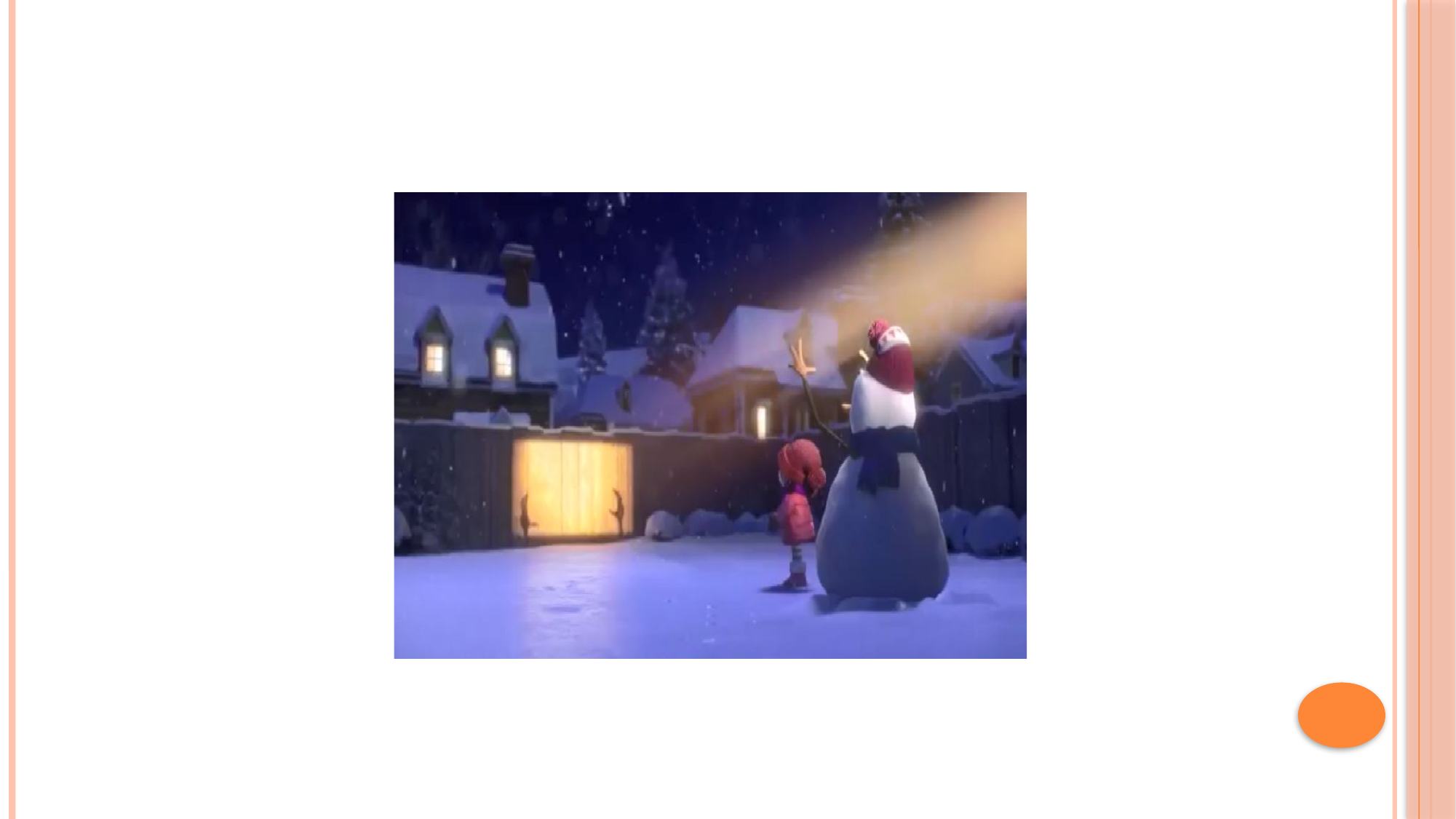

## Slide 10
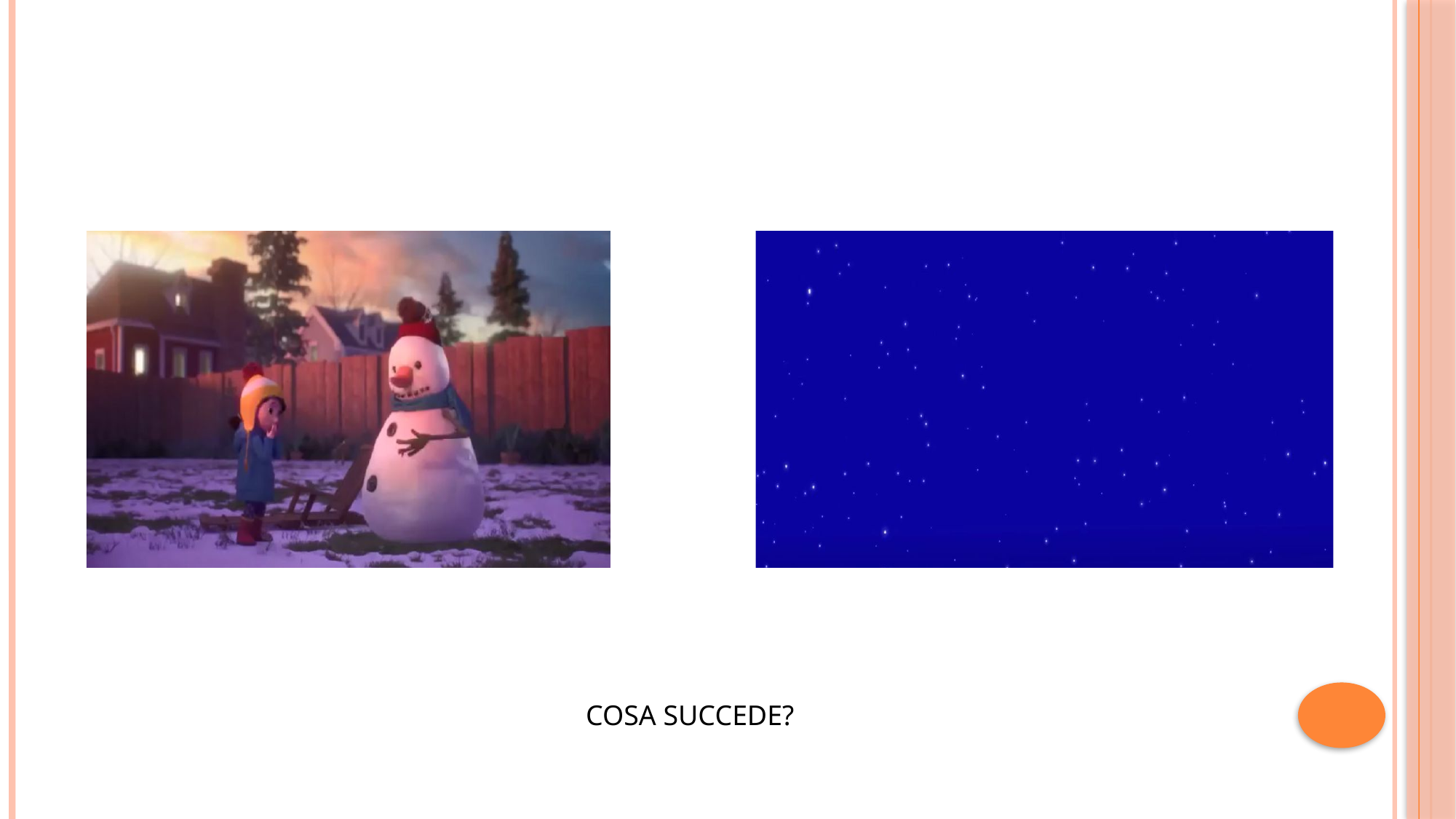

COSA SUCCEDE?

## Slide 11
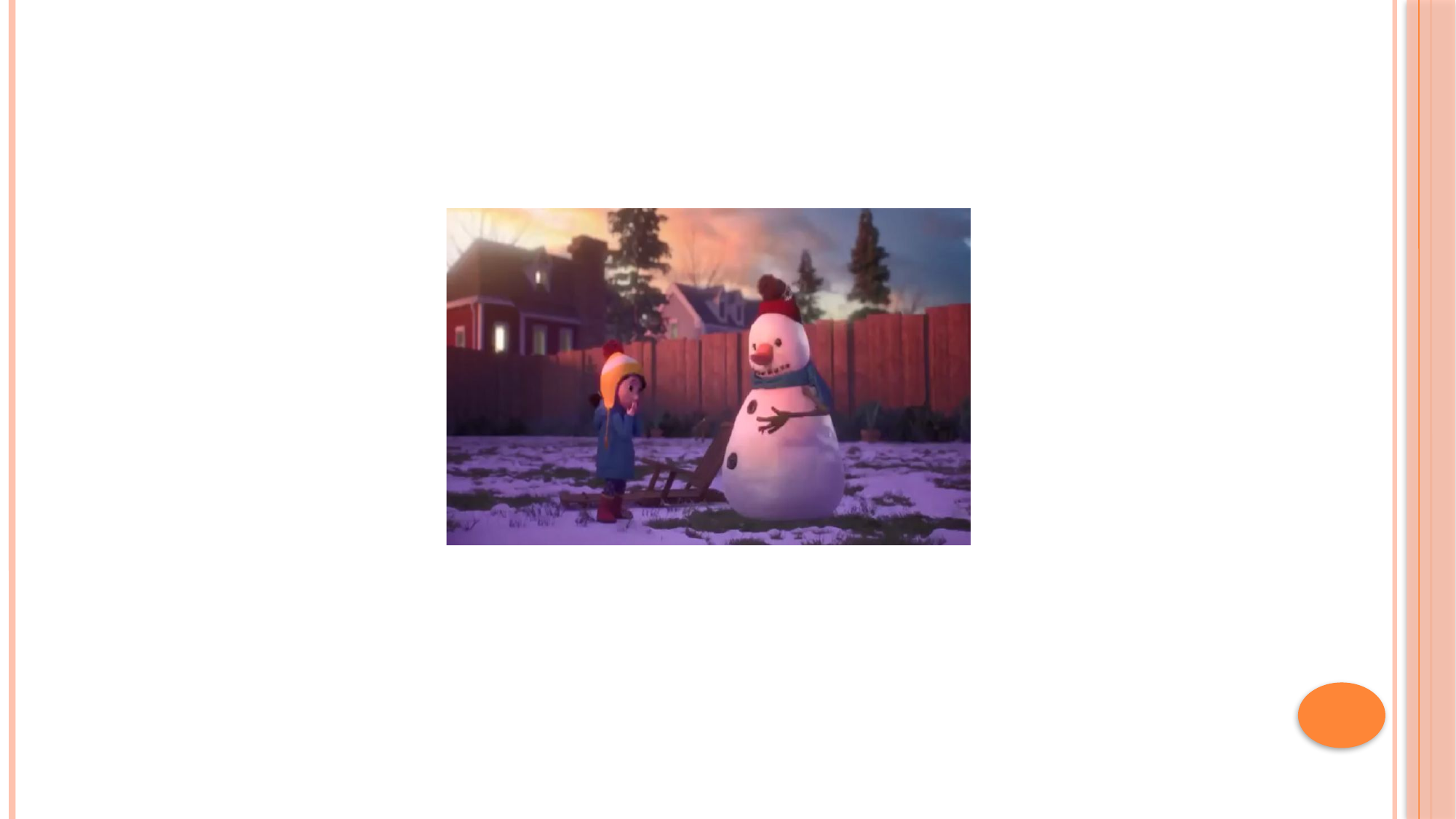

## Slide 12
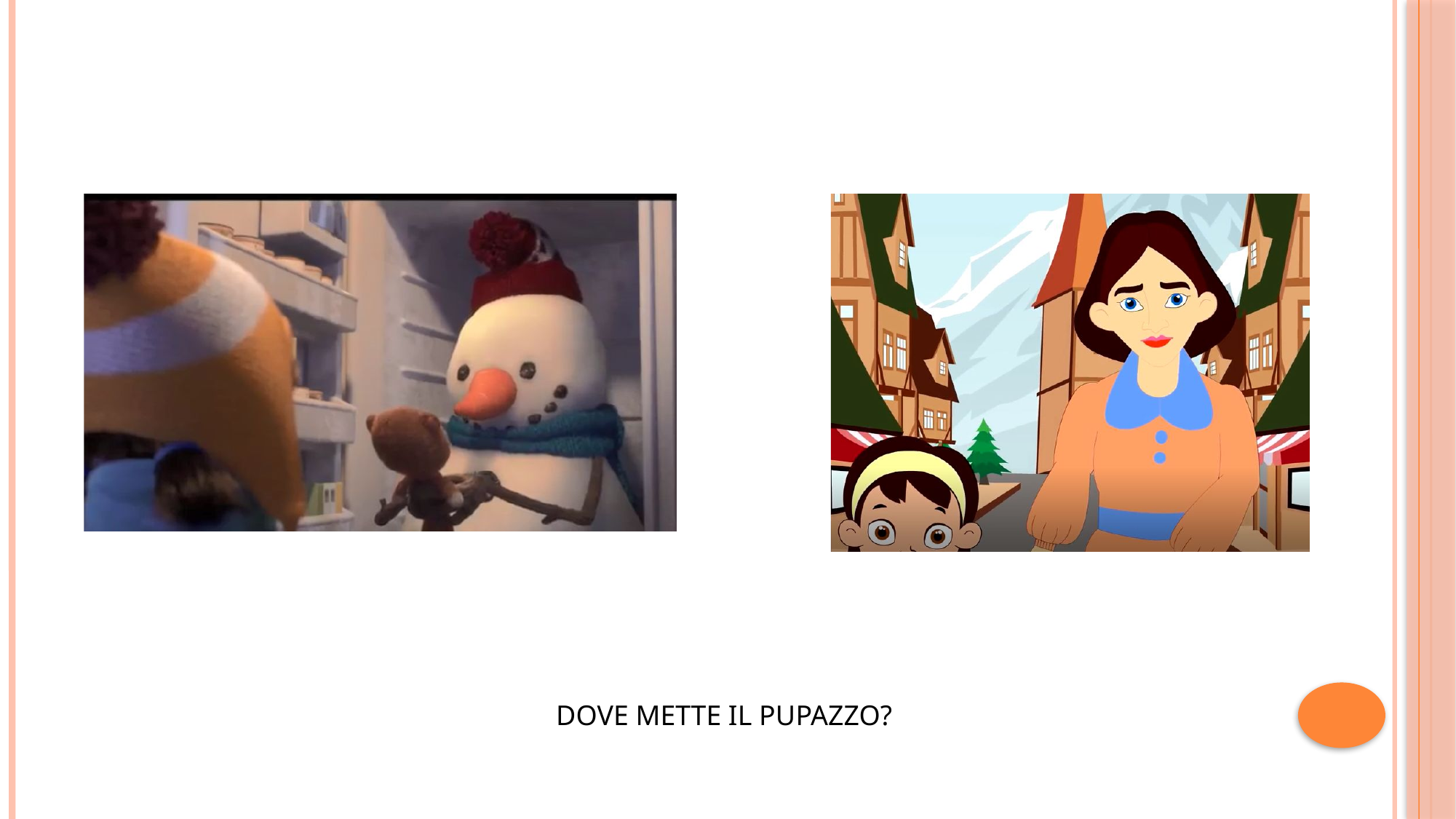

DOVE METTE IL PUPAZZO?

## Slide 13
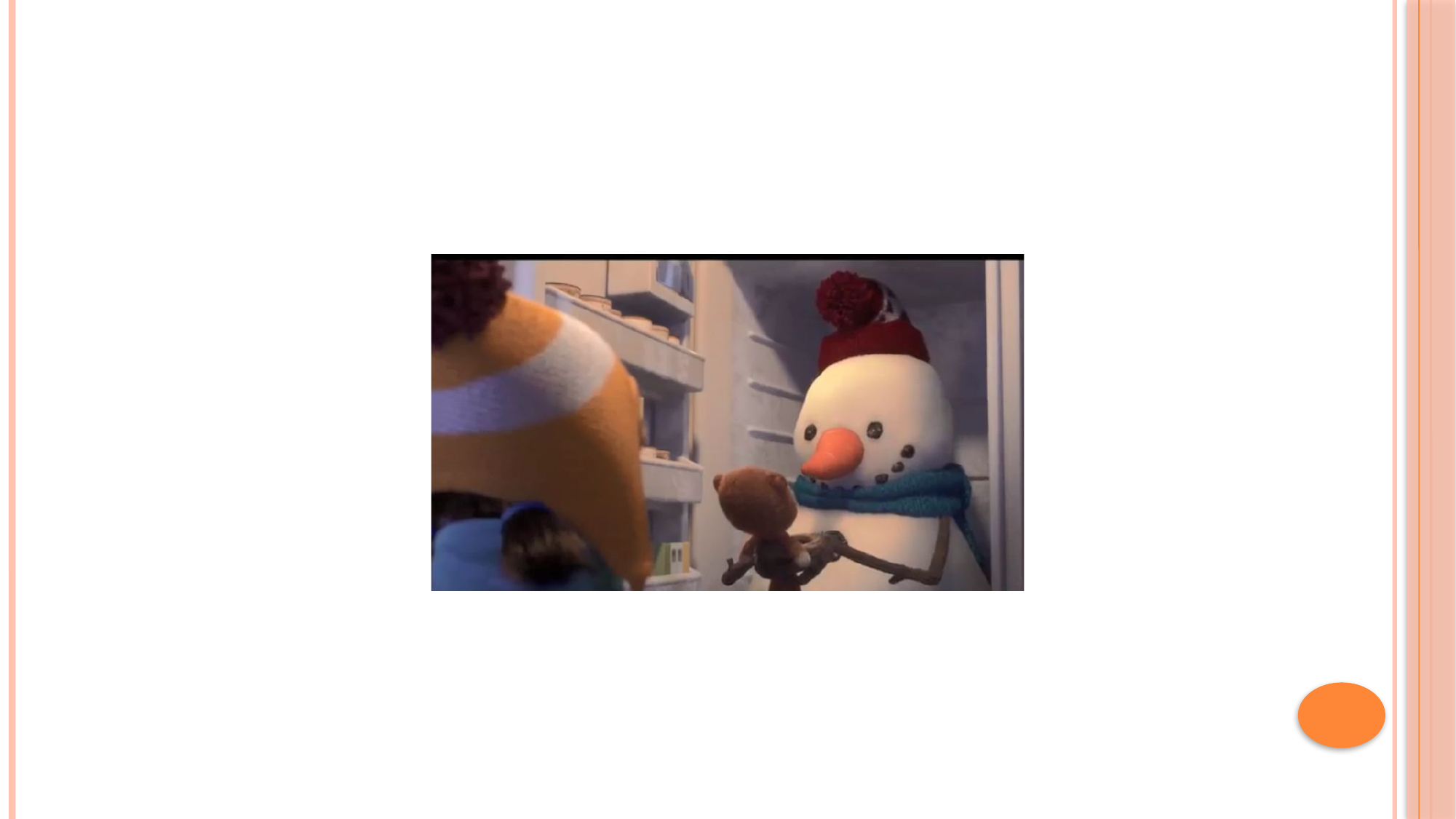

## Slide 14
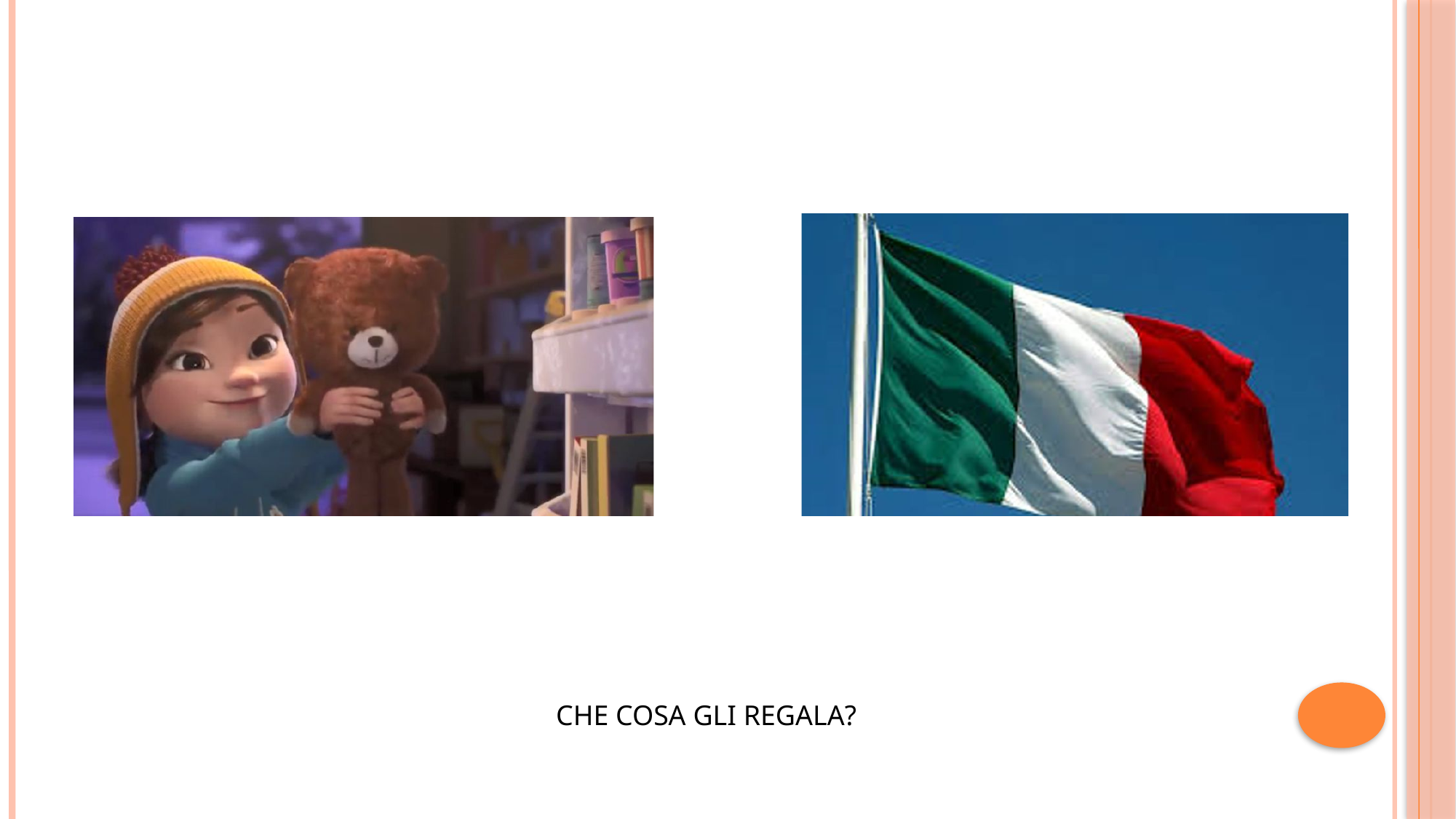

CHE COSA GLI REGALA?

## Slide 15
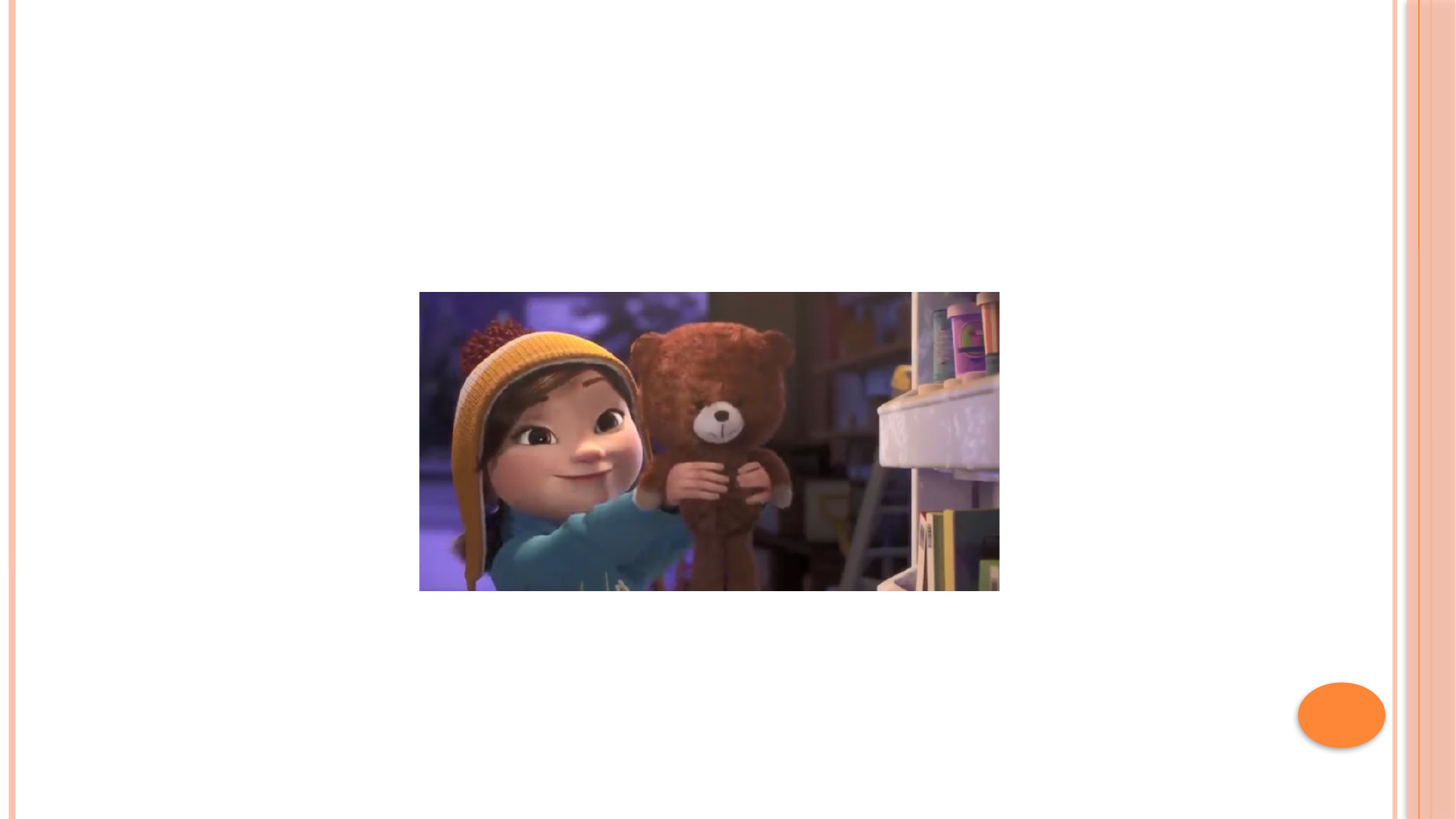

## Slide 16
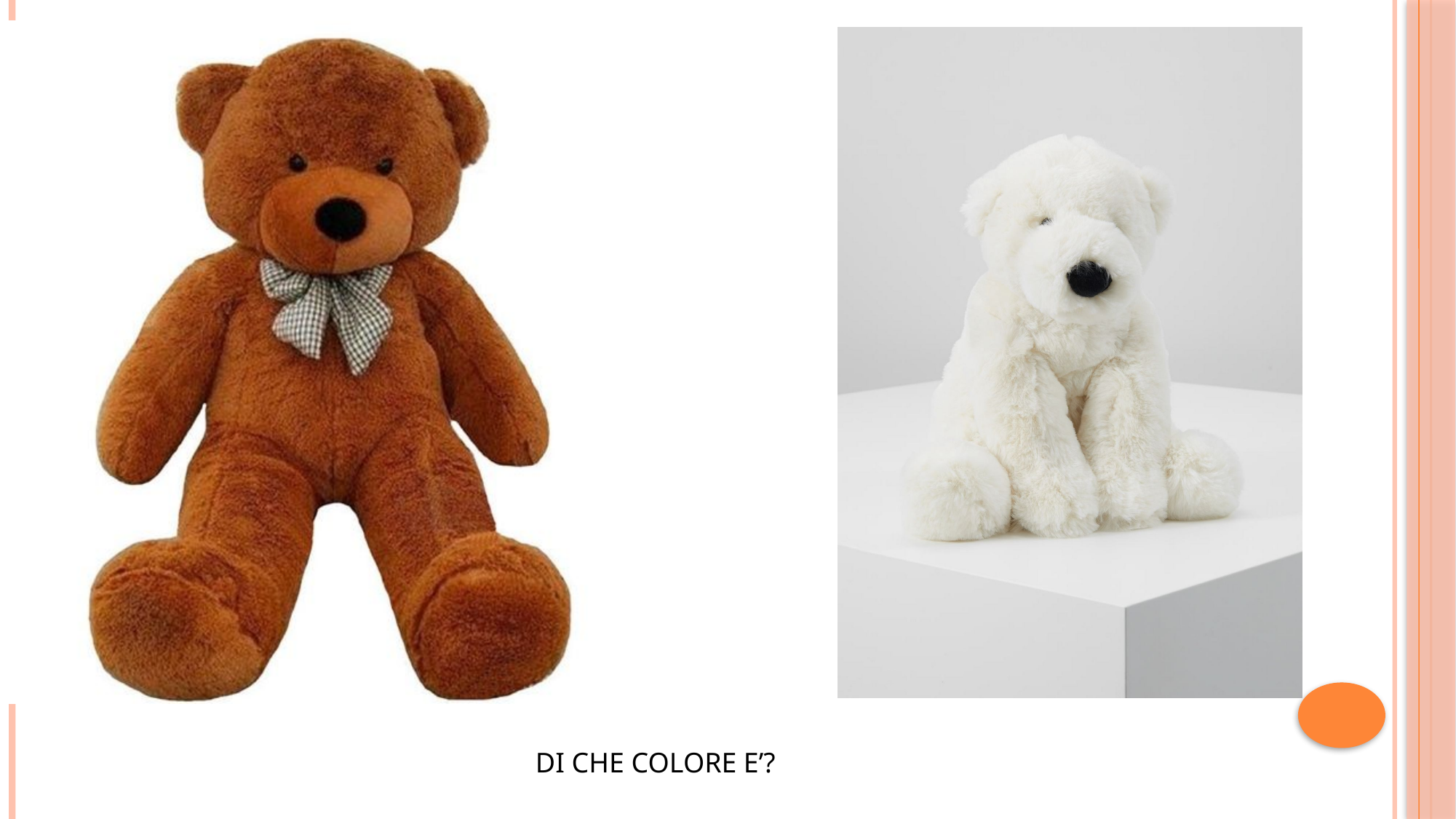

#
DI CHE COLORE E’?

## Slide 17
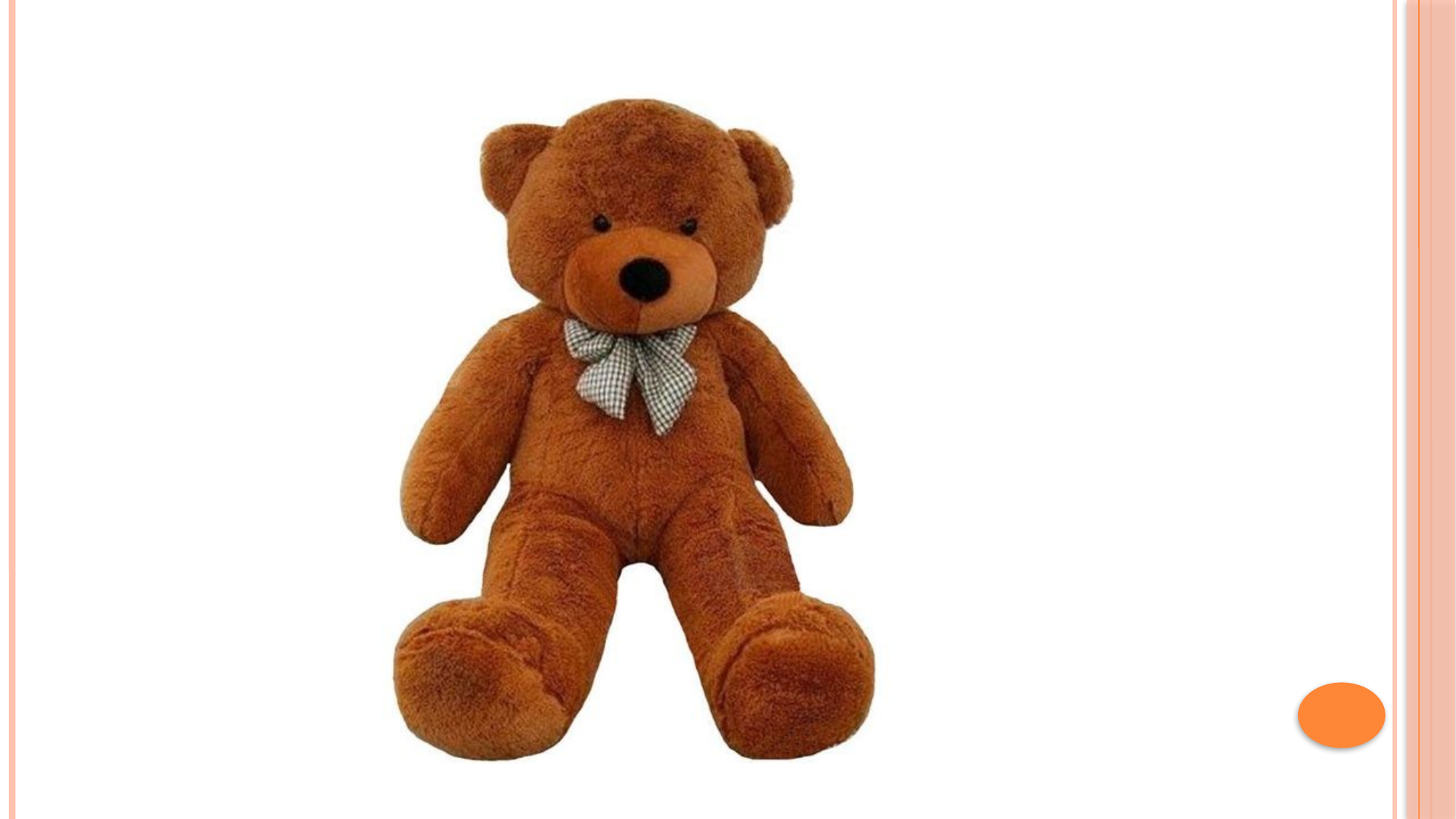

#

## Slide 18
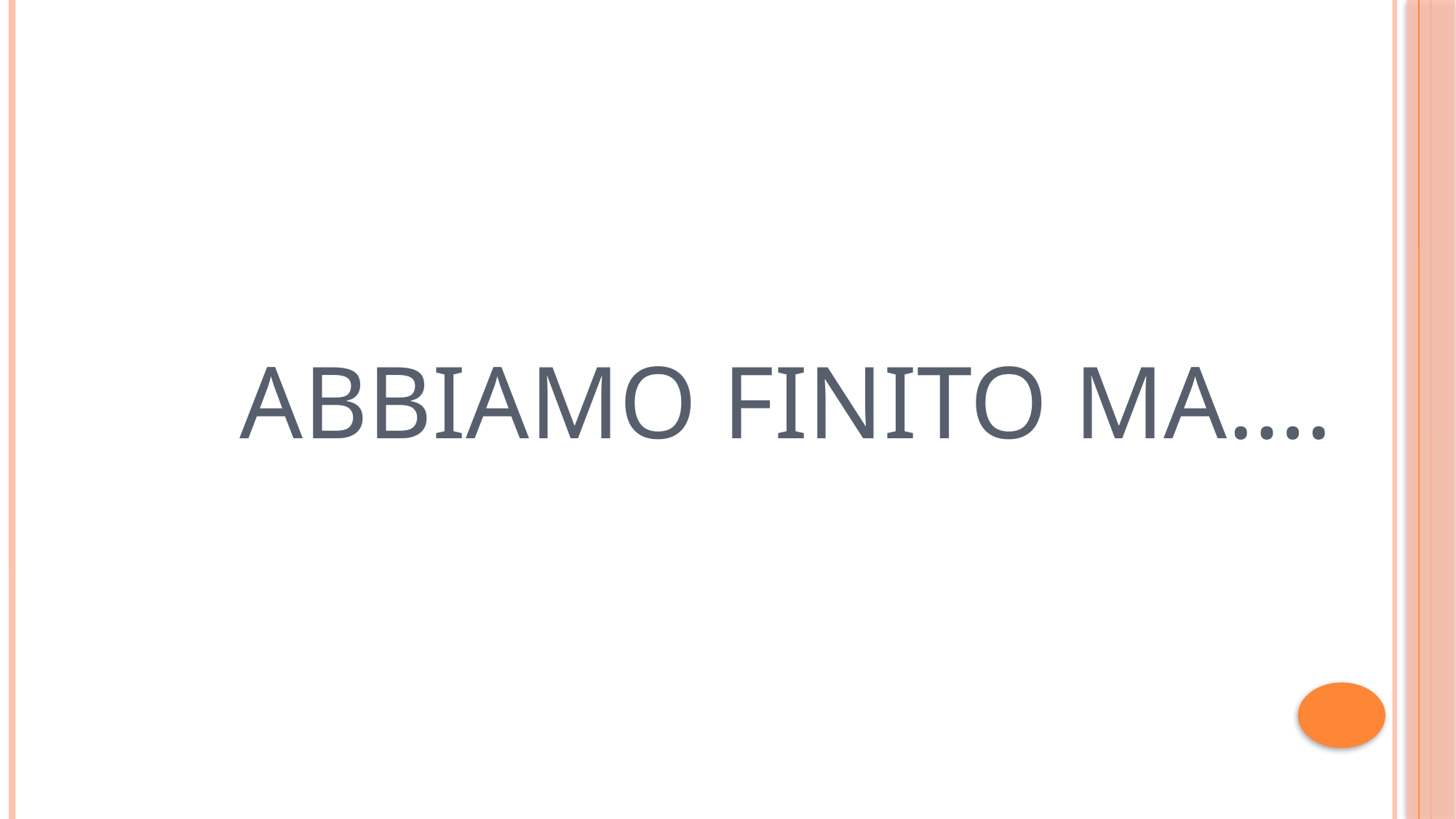

# Abbiamo finito ma….
