# Supplementary figures and images for: Social and Cognitive Interactions Through an Interactive School Service for RTT Patients at the COVID-19 Time
Source: Front Psychol. 2021 Jun 24;12:676238. doi: 10.3389/fpsyg.2021.676238 (PMC8265204; doi:10.3389/fpsyg.2021.676238)

## Slide 1
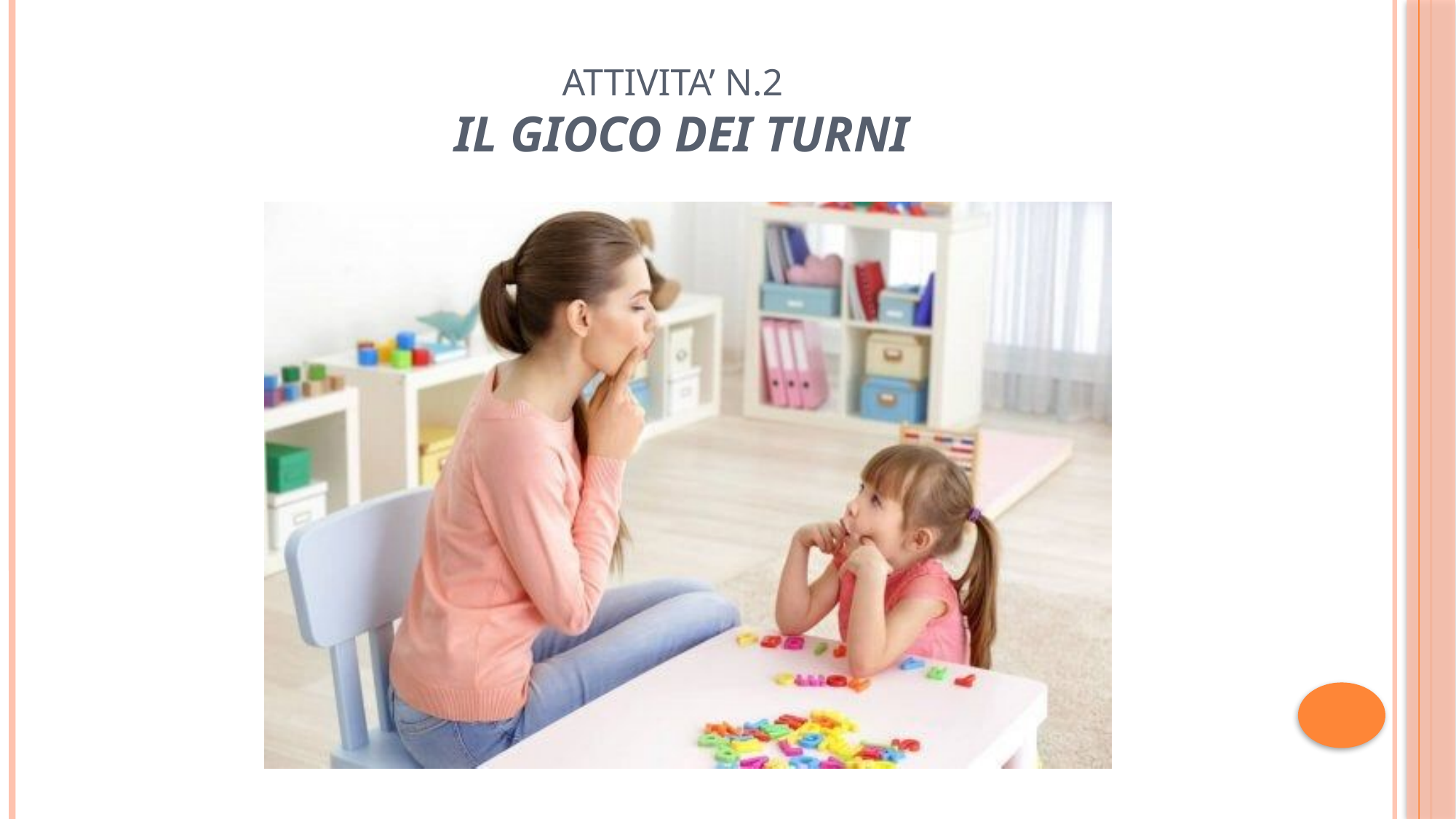

# ATTIVITA’ N.2 Il gioco dei turni

## Slide 2
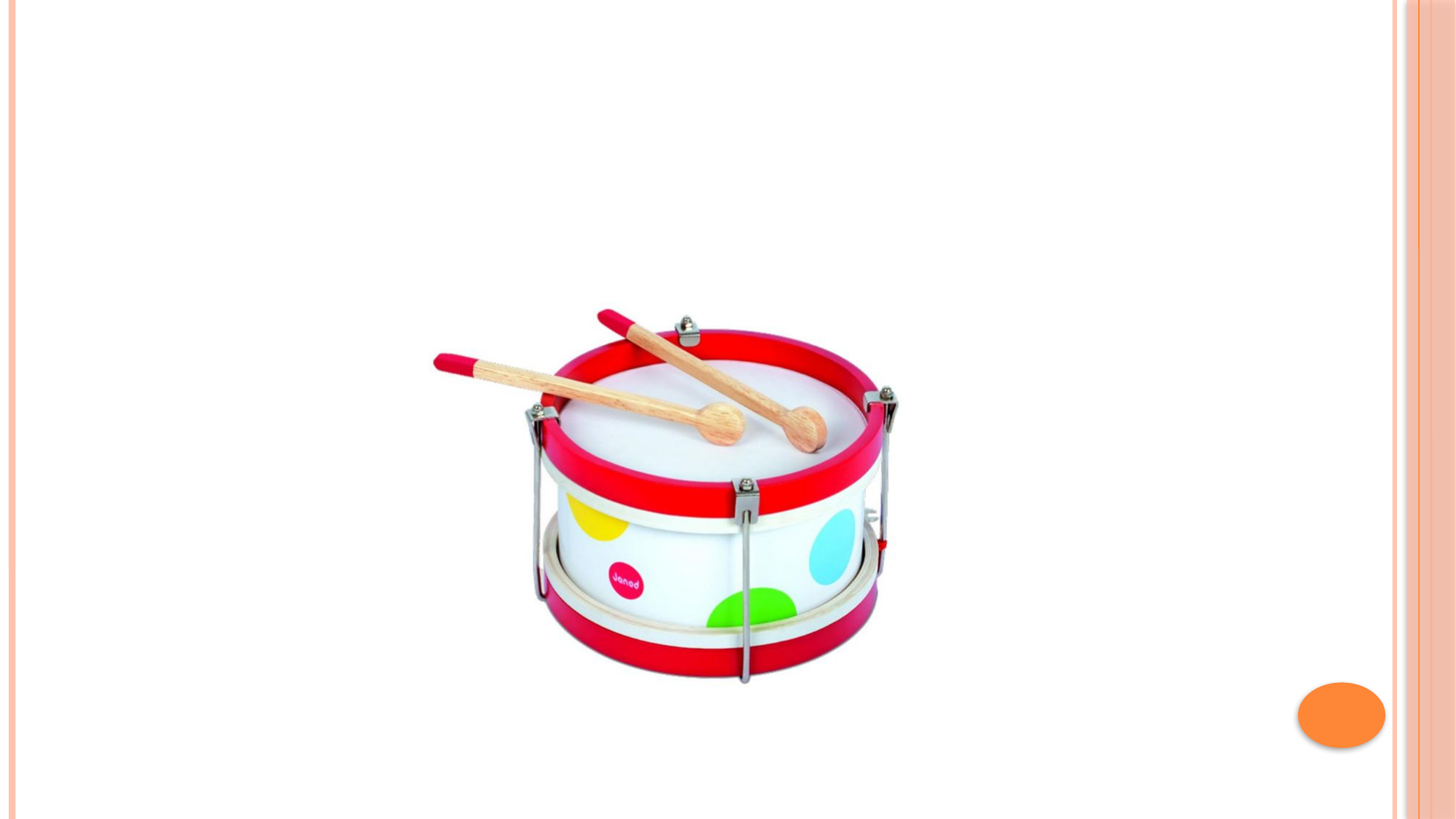

## Slide 3
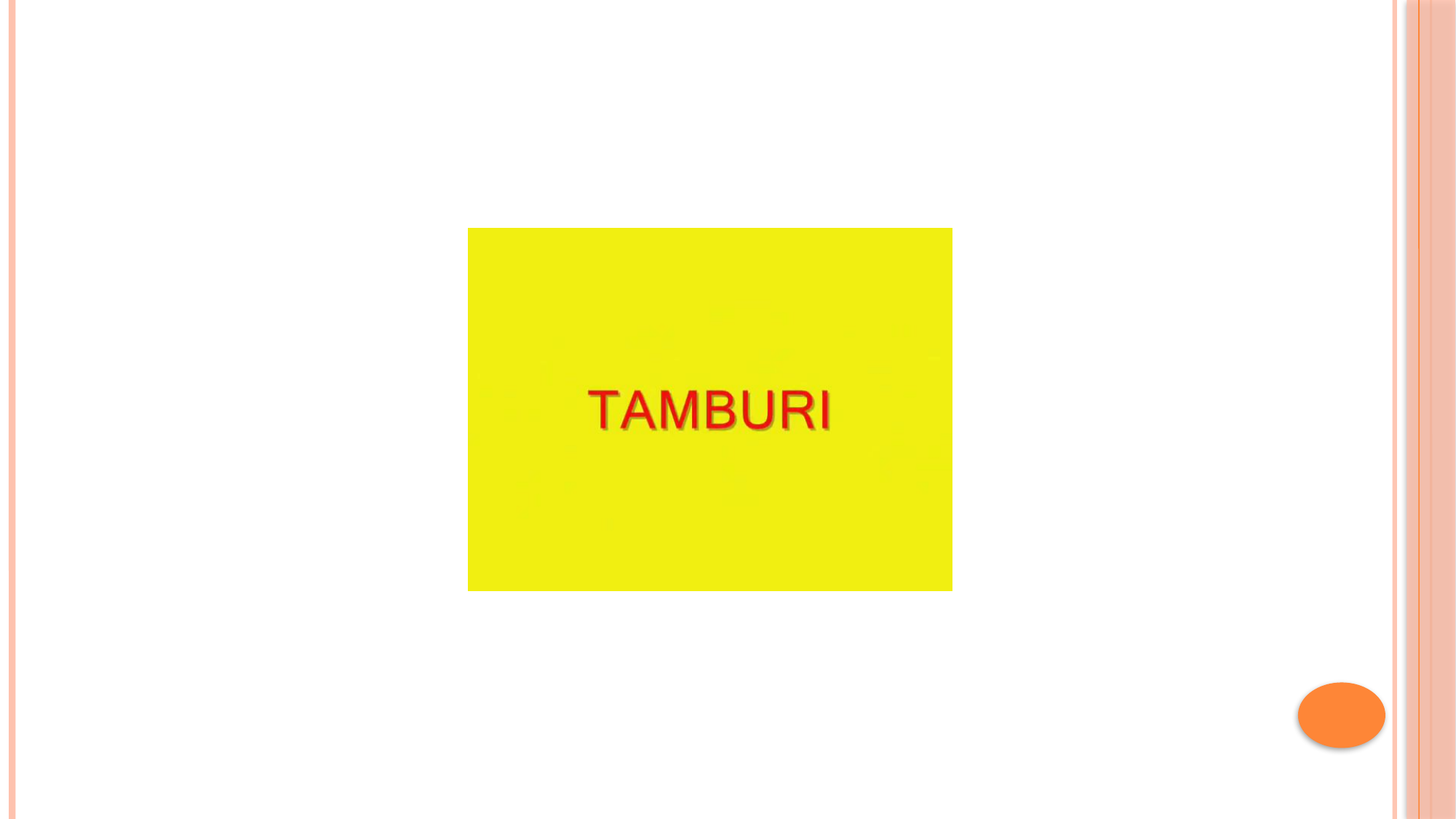

Supplement: Supplementary file 4 [file Presentation_2.pptx]
